# Dihydromyricetin resists inflammation‐induced muscle atrophy via ryanodine receptor‐CaMKK‐AMPK signal pathway

**DOI:** 10.1111/jcmm.16810

**Published:** 2021-10-22

**Authors:** Lianjie Hou, Fangyi Jiang, Bo Huang, Weijie Zheng, Yufei Jiang, Gengyuan Cai, Dewu Liu, Ching Yuan Hu, Chong Wang

**Affiliations:** ^1^ Guangdong Provincial Key Lab of Agro‐Animal Genomics and Molecular Breeding National Engineering Research Center for Breeding Swine Industry College of Animal Science South China Agricultural University Guangzhou China; ^2^ The Sixth Affiliated Hospital of Guangzhou Medical University Qingyuan City People's Hospital Qingyuan China; ^3^ Department of Human Nutrition, Food and Animal Sciences College of Tropical Agriculture and Human Resources University of Hawaii at Manoa Honolulu Hawaii USA

**Keywords:** dihydromyricetin, mice, obesity, skeletal muscle atrophy, target protein

## Abstract

Skeletal muscle plays a pivotal role in the maintenance of physical and metabolic health. Skeletal muscle atrophy usually results in physical disability, inferior quality of life and higher health care costs. The higher incidence of muscle atrophy in obese and ageing groups is due to increased levels of inflammatory factors during obesity and ageing. Dihydromyricetin, as a bioactive polyphenol, has been used for anti‐inflammatory, anti‐tumour and improving insulin sensitivity. However, there are no published reports demonstrated the dihydromyricetin effect on inflammation‐induced skeletal muscle atrophy. In this study, we first confirmed the role of dihydromyricetin in inflammation‐induced skeletal muscle atrophy in vivo and in vitro. Then, we demonstrated that dihydromyricetin resisted inflammation‐induced skeletal muscle atrophy by activating Ca^2+^‐CaMKK‐AMPK through signal pathway blockers, Ca^2+^ probes and immunofluorescence. Finally, we clarified that dihydromyricetin activated Ca^2+^‐CaMKK‐AMPK signalling pathway through interaction with the ryanodine receptor, its target protein, by drug affinity responsive target stability (DARTS). Our results not only demonstrated that dihydromyricetin resisted inflammation‐induced muscle atrophy via the ryanodine receptor‐CaMKK‐AMPK signal pathway but also discovered that the target protein of dihydromyricetin is the ryanodine receptor. Our results provided experimental data for the development of dihydromyricetin as a functional food and new therapeutic strategies for treating or preventing skeletal muscle atrophy.

## 1 INTRODUCTION

Skeletal muscle comprises about 45% of the human body mass. Obesity and ageing are often accompanied by skeletal muscle atrophy.^1^ The main feature of skeletal muscle atrophy is a reduction in muscle mass and myofibre diameter. Skeletal muscle atrophy usually results in physical disability, inferior quality of life, and higher healthcare costs.^2^ Since skeletal muscle plays an essential role in maintaining body flexibility, it is critical for us to understand the mechanism of skeletal muscle atrophy for maintaining optimal health throughout life.

The skeletal muscle mass depends on the balance between protein synthesis and degradation in myofibre.^3^ The main signalling pathways that regulate skeletal muscle growth include the insulin signalling pathway responsible for protein synthesis and the ubiquitin‐proteasome system responsible for protein degradation. Insulin activates the IRS‐1/PI3K/AKT pathway by binding to the insulin receptor. AKT activates the mammalian target of rapamycin (mTOR)/S6 protein kinase (S6K) pathway increasing protein synthesis.^4^ E3 ubiquitin protein ligase is a key enzyme of the ubiquitin‐proteasome system,it recognizes the substrate and labels it by ubiquitination. The proteasome will then degrade the labelled protein. Muscle ring finger protein 1 (MuRF1) and muscle atrophy F‐box (atrogin‐1) are two muscle‐specific E3 ligases. MuRF1 and atrogin‐1 induce hydrolysis of skeletal muscle structure proteins and myogenic differentiation transcription factors, leading to skeletal muscle atrophy.^5^

The increasing incidence of skeletal muscle atrophy is closely related to the sharp increase in the obese population over the world. Obesity is characterized by increased production of tumour necrosis factor‐alpha (TNF‐α) and other pro‐inflammatory factors.^6^ Previous studies have shown that TNF‐α activated the c‐Jun N‐terminal kinase (JNK) pathway, induced the insulin resistance in skeletal muscle. Insulin resistance reduced protein synthesis in skeletal muscle.^7^ Other studies have reported that TNF‐α activated inhibitor of nuclear factor‐kappa B (IκB)/nuclear factor‐kappa B (NF‐κB) directly induced the expression of MuRF1 and atrogin‐1.^8^ In insulin‐resistant individuals, the effect of AKT inhibition on forkhead proteins disappeared and ultimately increases the expression of MuRF1 and atrogin‐1.^9^ Since there is a close relationship between obesity‐induced inflammation and skeletal muscle atrophy, preventing the skeletal muscle inflammation response in obese individuals will be a new strategy for reducing the incidence of skeletal muscle atrophy.

Dihydromyricetin (DHM), also known as ampelopsis, is the main bioactive polyphenol in rattan tea. Rattan tea has been used for anti‐inflammatory in China and other Asian countries for several centuries.^10^ DHM exerted its anti‐inflammatory effect in rats through suppressing NF‐kB signalling in macrophage.^11^ DHM also improved physical performance at high altitude by maintaining mitochondrial biogenesis in skeletal muscle.^12^ DHM prevented skeletal muscle insulin resistance by inducing autophagy through the AMPK‐PGC‐1α‐Sirt3 signalling pathway.^13^ Currently, most DHM‐related research reported that DHM functions depend on its activation of AMPK, but AMPK is not the direct target of DHM in cells, and the target of DHM in cells is still unclear. To our knowledge, there is no published paper demonstrated the DHM effect on inflammation‐induced skeletal muscle atrophy.

We used a high fat diet (HFD)‐induced obese mice model to study inflammation‐induced skeletal muscle atrophy in vivo. Gavage DHM was used to determine DHM function in reducing the level of inflammation and inhibiting skeletal muscle atrophy in obese mice. C2C12 cells were treated with TNF‐α as a model of inflammation‐induced muscle atrophy in vitro. We have elucidated the molecular mechanism of DHM for preventing inflammation‐induced skeletal muscle atrophy in C2C12 cells using immunofluorescence, signal pathway blocking and drug affinity responsive target stability (DARTS). Our results provided not only experimental data for the development of DHM as a functional food but also provided new therapeutic strategies for the skeletal muscle atrophy.

## 2 MATERIAL AND METHODS

All animal studies have been approved by the appropriate ethics committee and have therefore been performed in accordance with the ethical standards laid down in the 1964 Declaration of Helsinki and its later amendments. All experiments were conducted according to “The Instructive Notions with Respect to Caring for Laboratory Animals” issued by the Ministry of Science and Technology of the People's Republic of China. All experimental protocols and methods were approved by the College of Animal Science, South China Agricultural University.

### 2.1 Animal experiment

Thirty‐six 18‐day‐old specific‐pathogen‐free (SPF) healthy male C57B/L6 mice were purchased from the Animal Experiment Center of Guangdong Province. The mice were housed under a 12‐h light and 12‐h dark cycle (7 am and 7 pm, 25℃ and 70% ~ 80% humidity). The mice were divided into two groups randomly: chow diet group (control, *n* = 12) and the HFD group (HFD, *n* = 24). Mice body weight gain was measured every Monday morning. After 8 weeks, the body composition of the mice, the strength of the skeletal muscles and the endurance exercise capacity of the mice were measured, to make sure the model of obesity‐induced skeletal muscle atrophy was successfully constructed. Then, the obese mice were divided into two groups randomly: the HFD + PBS gavage (HFD) and the HFD + DHM gavage (DHM, purity by HPLC ≥ 98%, Shanghai Standard Biotech Co., Ltd.). Two hundred microliters of DHM (200 mg/kg body weight) was administered orally by gavage to the DHM group daily, while the control group and the HFD group were administered the same volume of PBS each day. We chose the rational dose of DHM (200 mg/kg body weight) according to the literature.^14^, ^15^ The body weight (BW) and feed intake of the mice were analysed every week. At week 18, we analysed the body composition of mice and monitored the respiratory exchange rate and exercise frequency of mice in a metabolic cage. At week 19, we tested the mice's skeletal muscle grip strength and exercise endurance. At week 20, the mice were sacrificed to collect serum, gastrocnemius, tibialis anterior muscle and other tissues for further analysis.

### 2.2 Haematoxylin‐eosin staining (H&E)

The mice skeletal muscle was fixed in 4% formaldehyde (DaMao) at room temperature for 48 h. The method used for the H&E staining has been described previously.^16^ The muscle fibre diameter was measured using Image‐Pro Plus (IPP) 6.0 software (Media Cybernetics, Inc.).

### 2.3 Oxygen consumption and exercise frequency assay

After mice were administered oral gavage with DHM for 10 weeks, O_2_ consumption (VO_2_), respiratory exchange ratio (RER), and exercise frequency of the mice were obtained by the promotion metabolism measurement system (Sable Systems International, USA).

### 2.4 Strength and exercise endurance assay

The maximum muscle force measured 5 times by a grip strength meter (BIO‐GS3, Bioseb/France), and the mean maximum strength of the twelve mice was used for data analysis of muscle strength. The mice exercise endurance measured on the FT‐200 Animal treadmill (Techman) at a speed of 10 m/min. Keep the mice running to exhaustion and record the time.

### 2.5 Body composition analysis

Fat mass, lean mass, and body composition were determined using a nuclear magnetic resonance system according to the manufacturer's instruction (Body Composition Analyzer MiniQMR23‐060H‐I, Niumag Corporation).

### 2.6 C2C12 cell culture and inflammatory induce C2C12 cell muscle atrophy

The C2C12 cell line used in this study was purchased from American Type Culture Collection (ATCC). C2C12 cells were cultured in DMEM/HIGH GLUCOSE (Hyclone) with 10% foetal bovine serum (Gibco). C2C12 cells were seeded in 24‐well plates (4 × 10^5^ /cm^2^). After 24 h, we treated the C2C12 cells with TNF‐α (MedChemExpress, Monmouth Junction, USA) at the concentration of 1 ng/ml for 7 days to induce C2C12 cell muscle atrophy. Dissolve 3.2 mg DHM into 10 ml DMSO to prepare a 1 mM DHM solution. In C2C12 cell experiments, the DHM solution was mixed into the cell culture medium at a ratio of 1/1,000. We treated C2C12 cells with TNF‐α and DHM (1 μM, purity ≥ 98%, Sigma Chemical Inc.) for 7 days to demonstrate DHM resisted inflammation‐induced muscle atrophy. We treated C2C12 cells with TNF‐α, DHM and Compound C (5 μM, MedChemExpress) for 7 days to demonstrate DHM resisted inflammation‐induced muscle atrophy through AMPK. We treated C2C12 cells with TNF‐α, DHM and STO‐609 (10 ng/ml, MedChemExpress) for 7 days to demonstrate DHM‐resisted inflammation‐induced muscle atrophy through CaMKK. We treated C2C12 cells with TNF‐α, DHM and ryanodine (100 nM, MedChemExpress) for 7 days to demonstrate DHM resisted inflammation‐induced muscle atrophy through the ryanodine receptor.

### 2.7 Glucose uptake assay

Seven days after treatments, the glucose uptake was assayed by 2‐NBDG (MedChemExpress) according to the manufacturer's protocol. 2‐NBDG is a fluorescent glucose analog that has been used to monitor glucose uptake in live cells. Therefore, the intensity of 2‐NBDG immunofluorescence reflects glucose uptake and insulin sensitivity. C2C12 cells were incubated with or without media containing 10 nM insulin (Sigma Chemical Inc.) for 10 min. The media was changed to low‐glucose DMEM containing 150 μg/ml 2‐NBDG for 60 min at 37℃. The medium was removed, and cells were washed 5 times with PBS. Nikon Eclipse Ti‐s microscopy (Nikon) was used to observe fluorescence. Fluorescent data were acquired at excitation and emission wavelengths of 490 and intensity at 525 nm.

### 2.8 Methyl Thiazolyl diphenyl‐tetrazolium bromide (MTT)

C2C12 cells were seeded in 96‐well plates at a density of 3 × 10^4^ / cm^2^. After 12‐h culture, 15 µl of treatments (TNF or DHM) with different concentrations was added to the cells for another 24 h of incubation. MTT was performed according to the manufacturer's protocol (M1020‐500T, Solarbio).

### 2.9 Calcium (Ca^2+^) imaging

Ca^2+^ was measured by a Ca^2+^ fluorescent probe fluo‐4‐AM kit following the manufacturer's instructions. C2C12 cells were incubated with ryanodine (100 nM, MedChemExpress) or U73122 (1 μM, MedChemExpress, Monmouth Junction, USA) for 1 h to block the endoplasmic reticulum Ca^2+^ channel. Then, the cells were washed 3 times with Hank's balanced salt solution and incubated with 10 μM fluo‐4‐AM at 37℃ for 1 h. After incubation, cells were then rewashed 3 times. Nikon Eclipse Ti‐s microscopy (Nikon) was used to observe fluorescence. Fluorescent data were acquired after excitation at 490 nm and intensity at 525 nm.

### 2.10 RNA Extraction and PCR Analysis

Methods used for the RNA extraction and PCR analysis have been described previously.^17^ The relative expression of mRNAs was normalized with β‐actin levels using the 2^−ΔΔCt^ method. 2^−ΔΔCt^ is defined as the ratio of the relative mRNA or miRNA level between the experimental group and the control group. Primers were designed using Primer Premier 5 based on sequences of mice genes obtained from NCBI. All the primers used in this study are shown in Table 1.

**TABLE 1:** Primers used in this study

Gene name	Forward primer sequence (5′−3′)	Reversed primer sequence (5′−3′)
β‐Actin	TCGTACCACTGGCATTGTGAT	CGAAGTCTAGGGCAACATAG
AMPK	GTATGCTGGTCCAGAGG	AAAGGCTAATCACAGAAGG
NF‐κB	ATGCCAGTGAGAATGTATGC	ACGCAGGAGACGGAAGAAT
IRS−1	ATGTCGCCAGTGGGAGATT	CTTCGGCAGTTGCGGTATA
mTOR	CTTAGAGGACAGCGGGGAAG	TGGTTTCCTCATTCCGGCTC
Myosin	CCAGGGCTCAGGTAGACCTT	CCCGCTAAGGGTCTTCGTA
JNK	CCAGCACCCATACATCAAC	TTCCTCCAAATCCATTACCTCC
GLUT4	AGTATGTTGCGGATGCTATGG	CTGCTCTAAAAGGTAAGGTGT
Atrogin	CATCAGGAGAAGTGGATCTAT	GCTTCCCCCAAAGTGCAGTA
MuRF1	TGTTCTGGTAGGTCGTTTCCG	ATGCCGGTCCATGATCACTT
AMPK	GTATGCTGGTCCAGAGG	AAAGGCTAATCACAGAAGG

### 2.11 Western blot analysis

The method used for the Western blot analysis has been described previously.^18^ Band intensities were quantified by ImageJ software. The antibodies and their dilutions used in this study are listed in Table 2.

**TABLE 2:** Antibody information used in this study

Primary antibody	Clone	Company	Catalog No.	Dilution
RYR	Polyclonal	Bioss	bs−6305R	1:500
CaMKK	Polyclonal	Abbkine	ABP53531	1:1,000
p‐CaMKK	Polyclonal	Affinit	AF4487	1:1,000
LKB1	Polyclonal	Bioss	bs−3948R	1:2,000
AMPK	Polyclonal	Abbkine	ABP50650	1:1,000
p‐AMPK	Polyclonal	Abbkine	ABP50452	1:1,000
JNK	Polyclonal	ZEN BIO	380556	1:2,000
NF‐κB	Polyclonal	Bioss	bsm−33117 M	1:5,000
p‐NF‐κB	Polyclonal	Bioss	bs−3485R	1:500
MuRF1	Polyclonal	Bioss	bs−2539R	1:1,000
Atrogin−1	Polyclonal	Bioss	bs−2591R	1:1,000
IRS−1	Polyclonal	Abcam	341420	1:1,000
mTOR	Monoclonal	CST	# 2972S	1:2,000
p‐mTOR	Monoclonal	CST	# 5536S	1:2,000
GLUT4	Polyclonal	Abcam	ab109313	1:2,000
Myosin	Monoclonal	BOSTER	BM0096	1:500
β‐Actin	Monoclonal	Bioworld	BS6007 M	1:5,000

Secondary antibody	Conjugate Used	Company	Catalog No.	Dilution
Goat anti‐rabbit IgG	HRP	Bioss	bs−0295G	1:5,000
Goat anti‐rabbit IgG (H+L)	HRP	Bioworld	BS13278	1:50,000
Goat anti‐mouse IgG (H+L)	HRP	Bioworld	BS12478	1:50,000
Goat anti‐mouse IgG (H+L)	Cy3	Bioworld	BS10006	1:200
Goat anti‐rabbit IgG	Alexa Fluor 647	Bioss	bs−0295G‐AF647	1:200

### 2.12 Immunofluorescent staining and confocal microscopy

C2C12 cell immunofluorescence staining was conducted after 7 days of treatments. The method used for MyHC and atrogin‐1 immunofluorescent assay has been described previously.^19^ The fluorescence was observed using Nikon Eclipse Ti‐s microscopy (Nikon). The cell nuclei were stained for DAPI (Beyotime).

### 2.13 Drug affinity responsive target stability (DARTS) assay

Drug affinity responsive target stability experiments for identifying the targets of DHM were performed as previously reported.^20^ In brief, C2C12 cells were lysed and treated with DHM (10 nM or 1 μM) for 1 h at room temperature. Then, the mixture was digested with 0.01% protease for 30 min at room temperature. The digestion was stopped by directly add 5×SDS‐PAGE loading buffer and inactivation by boiling 5 min. Protein samples were separated with 8%–15% SDS‐polyacrylamide gels and analysed by Coomassie blue staining and Western blotting.

### 2.14 Statistical analysis

**FIGURE 1:**
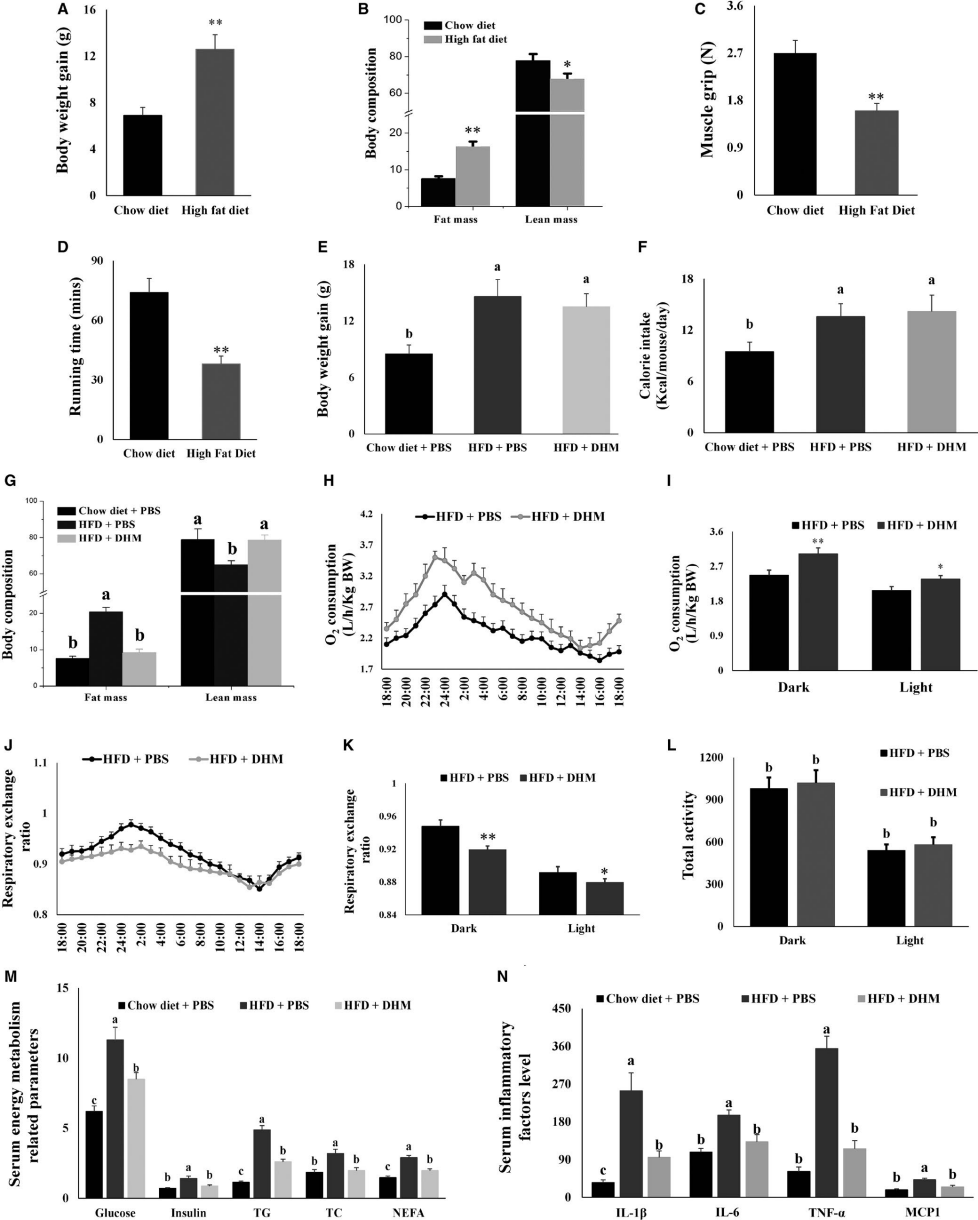
DHM (200 mg/kg body weight) reduced fat accumulation and inflammation in HFD‐induced obese mice. (A) HFD increased the weight gain of mice. (B) HFD changed the body composition of mice. (C) Skeletal muscle grip was decreased in obesity mice. (D) The endurance exercise ability of obese mice was decreased. (E) DHM did not affect HFD‐induced body weight gain in mice. (F) DHM did not affect HFD‐induced average daily energy intake. (G) DHM prevented HFD‐induced fat gain and muscle loss. (H) DHM increased oxygen consumption in mice during the 24 h monitoring period. (I) DHM increased accumulated oxygen consumption in mice during the day and night. (J) DHM reduced respiratory metabolic rate in HFD‐fed mice during the 24 h monitoring period. (K) DHM reduced accumulated respiratory metabolic rate in HFD‐fed mice during the day and night. (L) DHM did not change the frequency of exercise in mice. (M) DHM alleviated HFD‐induced high levels of glucose, insulin, total triglycerides, total cholesterol, and free fatty acids in the serum. (N) DHM reduced the increase of the HFD‐induced inflammatory factors in serum. *N* = 6, * *p* < 0.05, ** *p* < 0.01, Bars with different letters indicate they are significantly different (*p *< 0.05)

All data are expressed as the mean ± standard deviation (SD) of three independent experiments. Our data are a normal distribution, and the homogeneity of data between each group is equal under the SPSS analysis. In Figure 1A–D, I, K, L ^,^ Figure 3F and Figure 7E, unpaired Student's *t*‐test was used for p‐value calculations, where * is *p* < 0.05; and ** is *p* < 0.01. Significant differences among groups (≥3) were determined by one‐way ANOVA (SPSS v18.0, IBM Knowledge Center, Chicago, IL, USA). Multiple comparisons between the groups were performed using the S‐N‐K method. Bars with different letters indicate they are statistically significantly different (*p *< 0.05).

## 3 RESULTS

### 3.1 DHM reduced fat accumulation and inflammation in HFD‐induced obese mice

In the HFD‐induced obese stage, HFD significantly increased body weight gain in mice after eight weeks, compared with chow diet‐fed mice (Figure 1A). HFD also significantly increased body fat content in mice (Figure 1B), indicating that we have constructed HFD‐induced obese mice successfully. In addition, the muscle content was decreased in the HFD‐induced obese mice compared with chow diet‐fed mice (Figure 1B). Both muscle strength (Figure 1C) and endurance exercise capacity (Figure 1D) were lower in HFD‐induced obese mice compared with chow diet‐fed mice. These results indicated that we have successfully constructed an obesity‐induced skeletal muscle atrophy model.

In the DHM treatment stage, HFD increased energy intake and body weight gain in mice compared with the chow diet group, and DHM did not affect energy intake and body weight gain in our mice feeding experiments compared with the HFD group (Figure 1E, F). We also analysed the body composition of mice and found that HFD increased fat mass and decreased muscle mass compared with chow diet group (Figure 1G). Compared with the HFD group, DHM prevented the reduction of muscle mass caused by HFD (Figure 1G). Subsequently, we monitored the respiratory exchange rate and exercise frequency of mice in a metabolic cage. We found that DHM feeding did not change exercise frequency (Figure 1L) but increased oxygen consumption and decreased metabolic rate in mice compared with the chow diet‐fed group (Figure 1H–K). For the energy metabolism‐related parameters, HFD increased the levels of glucose, insulin, total triglycerides, total cholesterol and free fatty acids in serum compared with the chow diet group. DHM alleviated the increase of energy metabolism‐related parameters in serum caused by HFD compared with the HFD group (Figure 1J). Since obesity is always accompanied by low‐level inflammation, we also measured the level of the inflammatory factors in serum. HFD induced a higher level of IL‐1β, IL‐6, TNF‐α and MCP1 in obese mice compared with chow diet group, as measured by ELISA. DHM reduced the increase in the HFD‐induced inflammatory factors in serum compared with the HFD group (Figure 1K). These results indicated that DHM reduced fat accumulation and inflammation in HFD‐induced obese mice.

### 3.2 DHM resisted inflammatory‐induced skeletal muscle atrophy in mice

**FIGURE 2:**
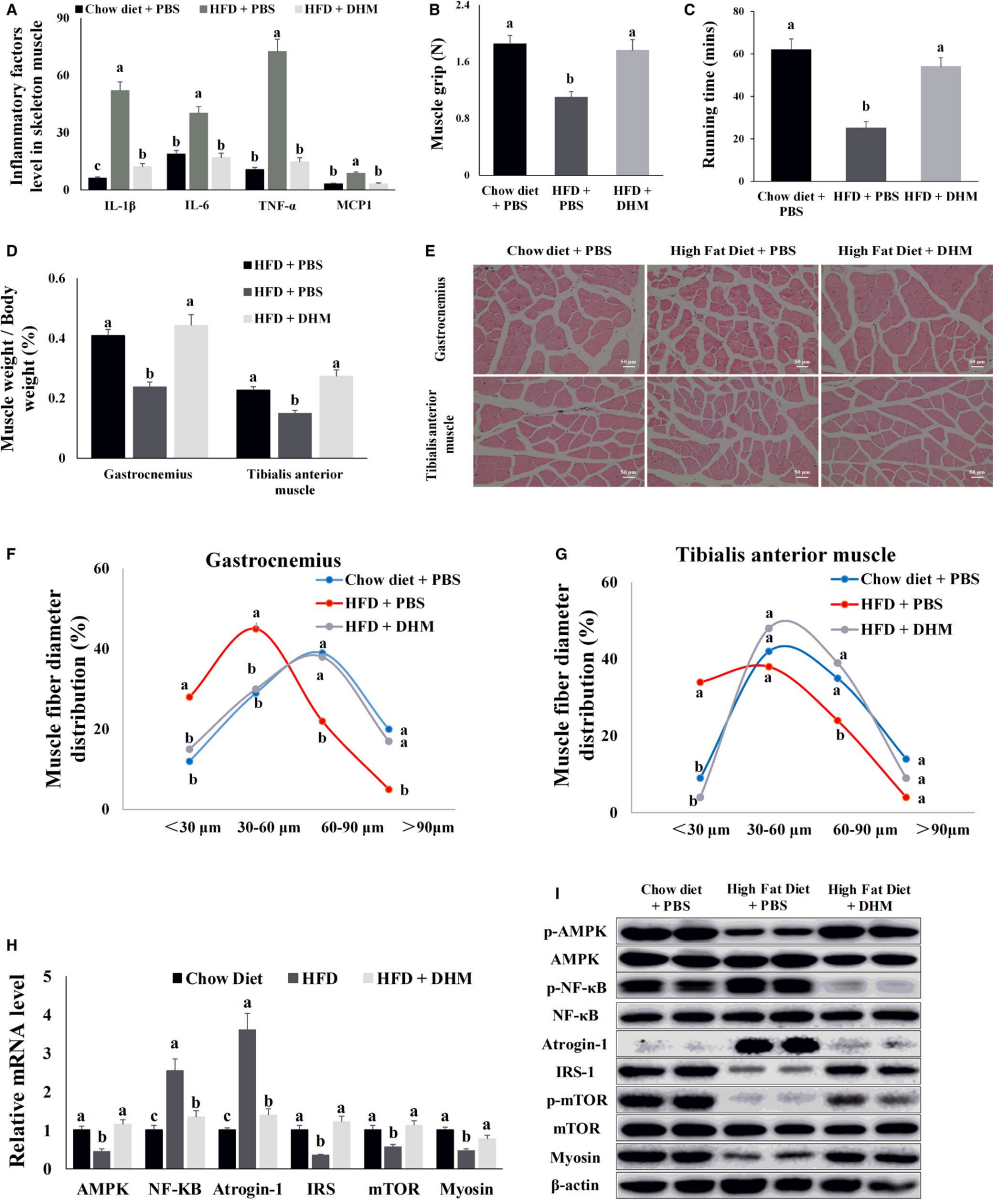
DHM (200 mg/kg body weight) resisted inflammation‐induced skeletal muscle atrophy in mice. (A) HFD elevated the level of skeletal muscle inflammatory factors in mice, and DHM suppressed the effect of HFD. (B) DHM enhanced skeletal muscle grip strength in obese mice. (C) DHM enhanced skeletal muscle exercise endurance in obese mice. (D) DHM inhibited skeletal muscle atrophy in obese mice, as shown by the percentage of muscle weight data. (E) HFD caused skeletal muscle atrophy in mice, and DHM prevented skeletal muscle atrophy in mice, as shown by H&E staining. (F) DHM prevented gastrocnemius muscle fibre atrophy. Muscle fibre diameter was measured using Image‐Pro Plus (IPP) 6.0 software. (G) DHM prevented atrophy of the tibialis anterior muscle fibres. Muscle fibre diameter was measured using Image‐Pro Plus (IPP) 6.0 software. (H) HFD inhibited the mRNA level of AMPK, IRS‐1, mTOR and myosin. HFD increased the mRNA level of NF‐κB and atrogin‐1. DHM reversed the mRNA level of inflammation‐induced muscle atrophy‐related genes. (I) HFD inhibited the protein level of p‐AMPK, IRS‐1, p‐mTOR, and myosin. HFD increased the protein level of p‐ NF‐κB and atrogin‐1. DHM reversed the protein level of inflammation‐induced muscle atrophy‐related genes. “p‐” before the gene name means phosphorylated form. *N* = 6, Bars with different letters indicate they are significantly different (*p *< 0.05)

To investigate whether DHM resisted inflammatory‐induced skeletal muscle atrophy, we measured the level of inflammatory factors and strength of the skeletal muscle in mice. ELISA results showed that HFD caused an increase in inflammatory factors IL‐1β, IL‐6, TNF‐α and MCP1 in skeletal muscle compared with the chow diet group. At the same time, DHM reduced the level of skeletal muscle inflammatory factors induced by HFD compared with the HFD group (Figure 2A). We also tested the mice's skeletal muscle grip strength and exercise endurance. Results from the skeletal muscle strength test showed that the grip strength and exercise endurance of obese mice decreased compared with the chow diet group, and DHM enhanced the skeletal muscle grip strength and exercise endurance of obese mice compared with the HFD group (Figure 2B, C). Both percentages of gastrocnemius weight/body weight and tibialis anterior muscle weight/body weight were decreased in HFD‐induced obese mice compared with the control group (Figure 2D). DHM reversed the HFD inhibitory effect on the gastrocnemius and tibialis anterior muscle weight gain compared with the HFD group (Figure 2D). The muscle fibre diameter of the gastrocnemius and tibialis anterior muscles were also reduced in the HFD‐induced obese mice based on the H&E results. DHM reversed the inhibitory effect of HFD on muscle fibre growth (Figure 2E–G). In addition, qPCR and Western blot results showed that AMPK, a skeletal muscle energy metabolism sensor, was inhibited in HFD‐induced obese mice compared with the chow diet group (Figure 2H, I and Figure S1). HFD‐induced obesity activated the expression of NF‐κB, the inflammatory response‐related gene; NF‐κB, then, in turn, induced the expression of atrogin‐1, the muscle atrophy‐related protein (Figure 2H, I and Figure S1). Finally, atrogin‐1 hydrolysed myosin, the skeletal muscle structural protein (Figure 2H, I and Figure S1). HFD‐induced obesity also decreased the expression of IRS‐1, indicating that HFD induced insulin resistance (Figure 2H, I and Figure S1). The insulin resistance, subsequently, inhibited the phosphorylation of mTOR, a marker gene for protein synthesis (Figure 2H, I and Figure S1). Expression of muscle atrophy‐related protein and inhibition of protein synthesis in muscle led to the skeletal muscle atrophy. However, DHM resisted inflammation‐induced skeletal muscle atrophy in mice by inhibiting the inflammation reaction and expression of muscle atrophy‐related proteins and enhancing insulin sensitivity and protein synthesis compared with the HFD group (Figure 2H, I and Figure S1).

### 3.3 TNF‐α induced inflammatory response and muscle atrophy in C2C12 cells

**FIGURE 3:**
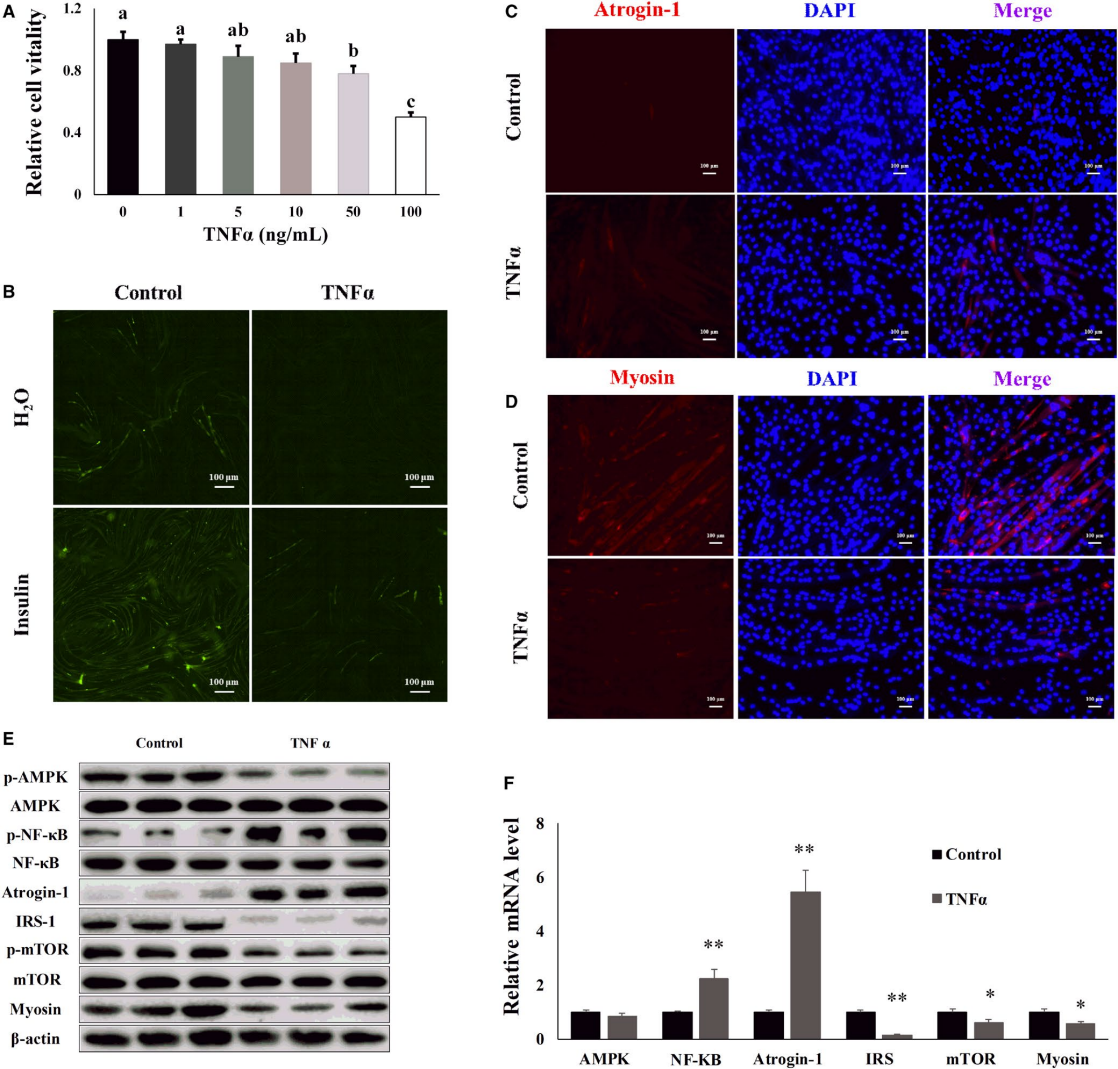
TNF‐α induced inflammatory response and muscle atrophy in C2C12 cells. (A) The optimal TNF‐α concentration used in C2C12 cell experiments was determined by MTT. (B) The glucose uptake test confirmed that TNF‐α induced insulin resistance in C2C12 cells. (C) Atrogin‐1 was visualized by the immunofluorescent assay; blue represents the nucleus, and red represents atrogin‐1. (D) Myosin was visualized by immunofluorescent assay; blue represents the cell nucleus; and red represents myosin. (E) WB detected the protein level of inflammation‐induced muscle atrophy‐related genes. “p‐” before the gene name means phosphorylated form. (F) mRNA level of inflammation‐induced muscle atrophy‐related genes was determined by qPCR. *N* = 6, * *p* < 0.05, ** *p* < 0.01, Bars with different letters indicate they are significantly different (*p *< 0.05)

We constructed a cellular model of TNF‐α‐induced muscle atrophy in C2C12 cells to establish the mechanism of DHM in relieving HFD‐induced inflammation and muscle atrophy. TNF‐α, less than 10 ng/ml, did not damage cell viability using MTT assay compared with the control group (Figure 3A). To simulate the low‐level inflammatory response during obesity, we selected the TNF‐α concentration of 1 ng/ml for 5 days in the subsequent experiments. After 5 days of TNF‐α treatment, insulin stimulation did not increase glucose uptake in C2C12 cells, and the cells developed an insulin resistance phenotype (Figure 3B and Figure S2A). Then, we measured the expression of astrogin‐1, a muscle atrophy marker and myosin, a skeletal muscle differentiation marker by immunofluorescence. TNF‐α treatment increased (*p* < 0.05) the expression of astrogin‐1 (Figure 3C and Figure S2B) and inhibited (*p* < 0.05) the expression of myosin compared with control group (Figure 3D and Figure S2C). Compared with the control group, the phosphorylation of AMPK was reduced, and the phosphorylation of NF‐κB was increased after TNF‐α treatment, according to the Western blot results (Figure 3E and Figure S2D). The phosphorylation of NF‐κB induced the expression of atrogin‐1, an atrophy‐related gene. TNF‐α treatment also decreased IRS‐1 expression compared with the control group, an insulin sensitivity‐related gene (Figure 3E, F and Figure S2D). The insulin resistance inhibited the phosphorylation of mTOR and inhibited myosin expression (Figure 3E, F and Figure S2D). Thus, the above results indicated that TNF‐α activated the inflammatory response and induced muscle atrophy. At the same time, the inflammatory response caused insulin resistance to inhibit protein synthesis and eventually exacerbated muscle atrophy.

### 3.4 DHM blocked expression of the inflammatory response‐induced muscle atrophy‐related genes

**FIGURE 4:**
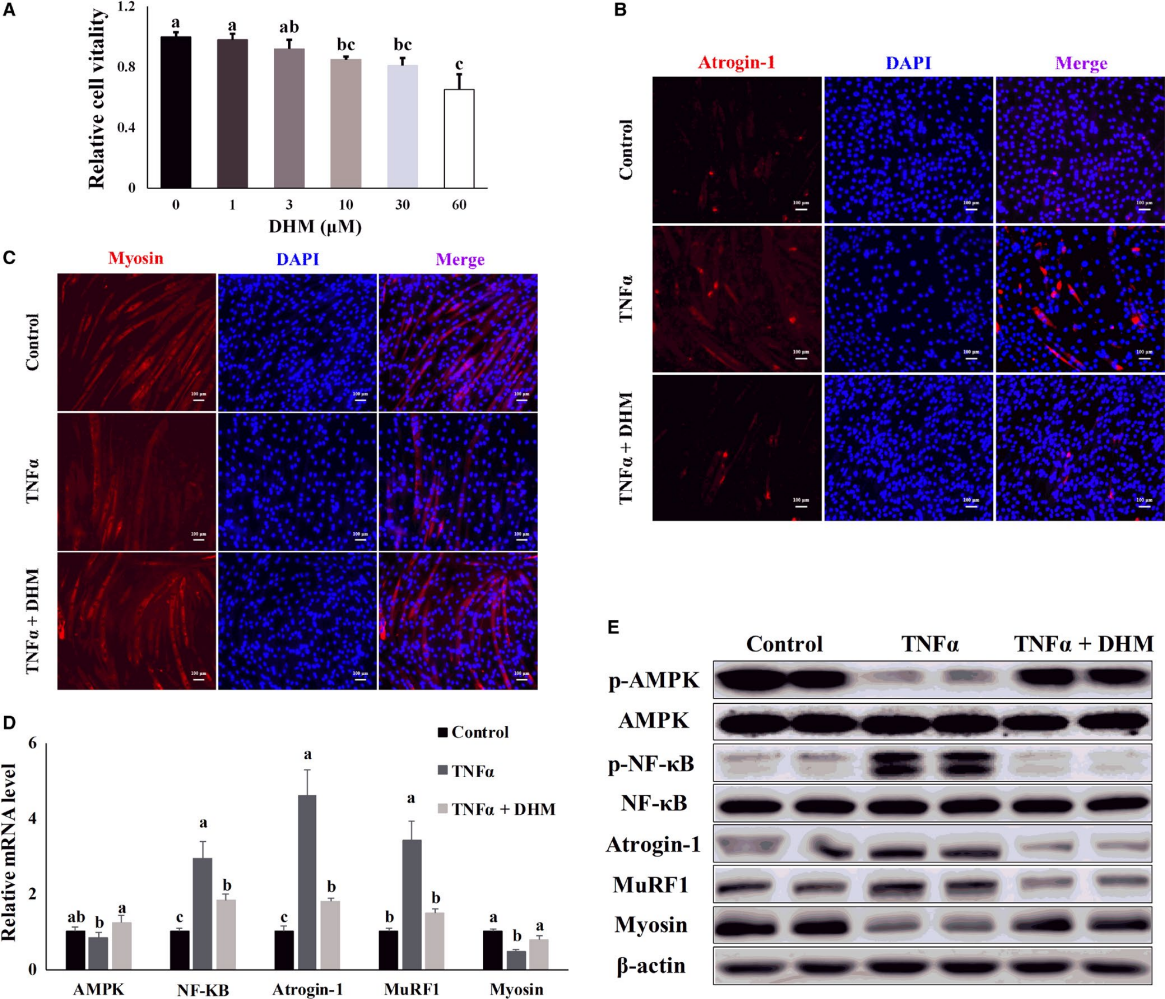
DHM (3 μM) blocked expression of the inflammatory response‐induced muscle atrophy‐related genes. (A) The optimal DHM concentration used in C2C12 cells was determined by MTT. (B) Atrogin‐1 was visualized by the immunofluorescent assay; blue represents the nucleus; and red represents atrogin‐1. (C) Myosin was visualized by immunofluorescent assay; blue represents the cell nucleus; and red represents myosin. (D) DHM effect on the mRNA level of the inflammatory response‐induced muscle atrophy‐related genes was detected by qPCR. (E) DHM effect on the protein level of the inflammatory response‐induced muscle atrophy‐related genes was detected by WB. “p‐” before the gene name means phosphorylated form. *N* = 6. Bars with different letters indicate they are significantly different (*p *< 0.05)

Then, we investigated whether DHM prevented inflammatory response‐induced muscle atrophy. Compared with the control group, DHM, less than 3 μM, did not damage cell viability through the MTT assay (Figure 4A). DHM treatment inhibited TNF‐α‐induced atrogin‐1 expression (Figure 4B and Figure S3A), and DHM reversed the inhibitory effect of TNF‐α on myogenic development compared with TNF‐α group, as shown by the immunofluorescence results (Figure 4C and Figure S3B). Compared with TNF‐α treatment, co‐treatment with TNF‐α and DHM, AMPK was activated, and NF‐κB was inhibited, as shown by the qPCR and WB results (Figure 4D, E and Figure S3C). Compared with TNF‐α treatment, DHM inhibited the expression of atrogin‐1 and MuRF1, two atrophy‐related genes. The inhibition of atrogin‐1 and MuRF1 inhibited the degradation of myosin (Figure 4D, E and Figure S3C). Based on these results, we conclude that DHM exerted its anti‐muscle atrophy function by blocking the expression of inflammation‐induced muscle atrophy‐related genes.

### 3.5 DHM improved TNF‐α‐induced insulin resistance and promoted protein synthesis in C2C12 cells

**FIGURE 5:**
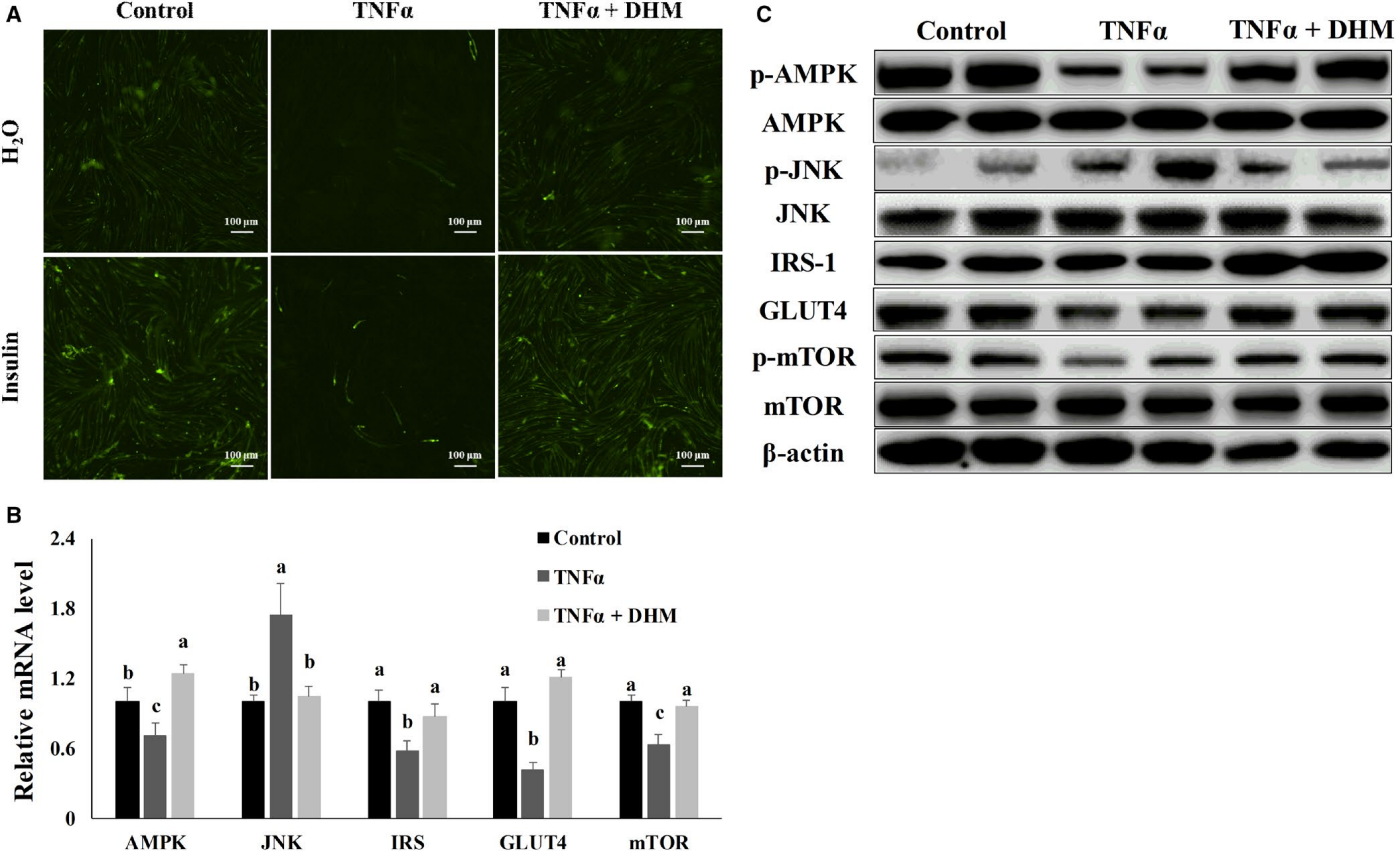
DHM (3 μM) improved TNF‐α‐induced insulin resistance and promoted protein synthesis in C2C12 cells. (A) Glucose uptake test confirmed that DHM relieved TNF‐α‐induced insulin resistance in C2C12 cells. (B) The mRNA level of the genes related to insulin signalling pathways and protein synthesis was determined by qPCR. (C) The protein level of the genes related to insulin signalling pathways and protein synthesis was measured by Western blot. “p‐” before the gene name means phosphorylated form. *N* = 6. Bars with different letters indicate they are significantly different (*p *< 0.05)

Improving myoblast insulin sensitivity and increasing myoblast protein synthesis also inhibited muscle atrophy. We then investigated whether DHM improved insulin resistance caused by TNF‐α. Sure enough, DHM increased insulin sensitivity and increased glucose uptake compared with the TNF‐α group (Figure 5A and Figure S4A). Compared with TNF‐α treatment, DHM increased the phosphorylation of AMPK, inhibited the phosphorylation of JNK, insulin resistance inducing factor and enhanced the expression of IRS‐1, insulin signalling pathway gene in the TNF‐α and DHM co‐treatment group (Figure 5B, C and Figure S4B). Activation of the insulin pathway by DHM increased the expression of glucose transporter GLUT4. Compared with TNF‐α treatment, DHM also stimulated the phosphorylation of mTOR, key gene in protein synthesis pathway (Figure 5B, C and Figure S4B). Based on these results, we conclude that DHM improved insulin resistance caused by TNF‐α, promoted protein synthesis in C2C12 cells and finally exerted DHM function of resisting inflammation‐induced muscle atrophy.

### 3.6 DHM resisted inflammation‐induced muscle atrophy through AMPK

**FIGURE 6:**
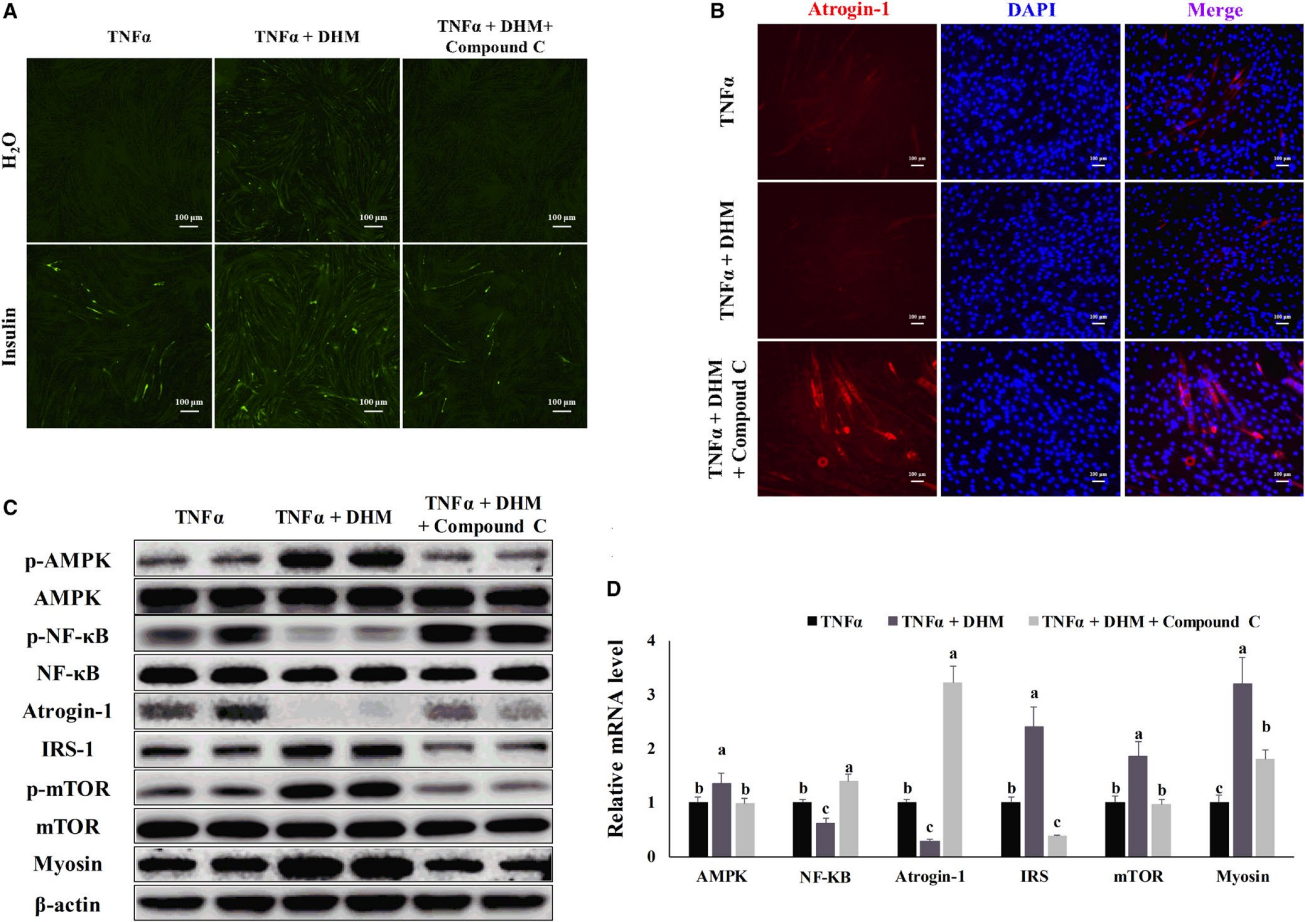
DHM (3 μM) resisted inflammation‐induced muscle atrophy through AMPK. (A) DHM alleviated TNF‐α‐induced insulin resistance was dependent on AMPK, as shown by the glucose uptake test. (B) DHM alleviated TNF‐α‐induced muscle atrophy was dependent on AMPK, as shown by the immunofluorescence results. Blue represents the nucleus, and red represents atrogin‐1. (C) DHM regulated the protein level of inflammatory response‐induced muscle atrophy‐related genes was dependent on AMPK, as shown by WB. (D) DHM regulated the mRNA level of inflammatory response‐induced muscle atrophy‐related genes was dependent on AMPK, as shown by qPCR. *N* = 6. Bars with different letters indicate they are significantly different (*p *< 0.05)

We blocked AMPK activity using Compound C to investigate whether DHM exerted its anti‐muscle atrophy function through AMPK in C2C12 cells. Compound C (5 μM) and DHM (3 μM) did not damage cell viability using MTT assay compared with the TNF‐α group (Figure S5D). The ability of DHM to improve TNF‐α‐induced insulin resistance was blocked by inhibiting AMPK, as shown by the glucose uptake assay (Figure 6A and Figure S5A). The ability of DHM to prevent TNF‐α‐induced muscle atrophy disappeared after blocking AMPK, as demonstrated by the immunofluorescence results (Figure 6B and Figure S5B). When Compound C blocked AMPK activity, the activation effect of DHM on AMPK and mTOR disappeared (Figure 6C and Figure S5C), and the promotion effect of DHM on the expression of IRS and myosin also disappeared (Figure 6C, D and Figure S5A). Blocked AMPK also caused DHM to lose its inhibitory effect on the NF‐κB (Figure 6C, D and Figure S5C); the DHM inhibitory effect on atrogin‐1 expression was also eliminated (Figure 6C, D and Figure S5C). These results indicated that DHM resisted inflammation‐induced muscle atrophy in C2C12 cells through AMPK.

### 3.7 DHM resisted inflammation‐induced muscle atrophy through CaMKK‐AMPK instead of LKB1‐AMPK pathway

**FIGURE 7:**
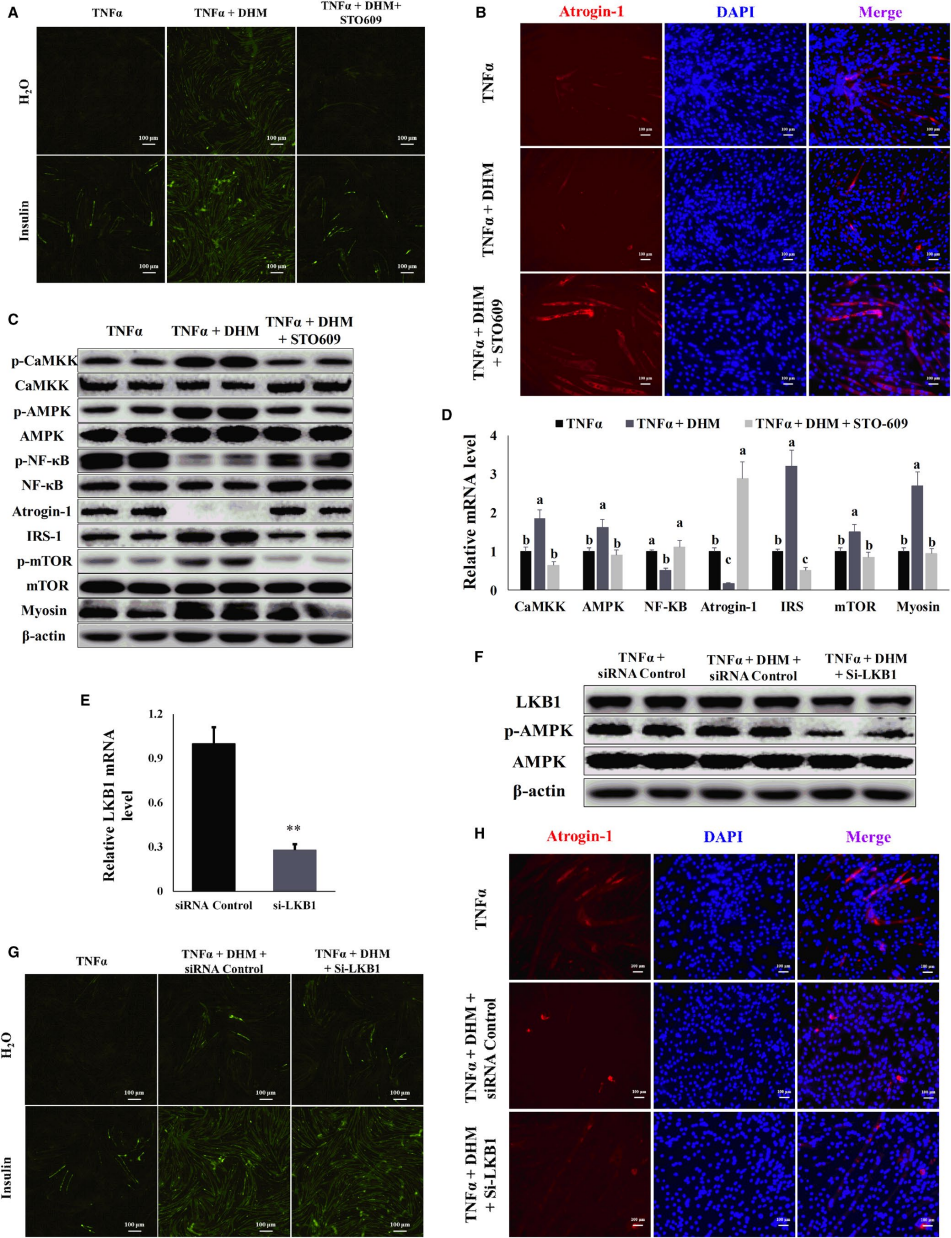
DHM (3 μM) resisted inflammation‐induced muscle atrophy through CaMKK‐AMPK instead of the LKB1‐AMPK pathway. (A) DHM alleviated TNF‐α‐induced insulin resistance was dependent on CaMKK, as shown by the glucose uptake test. (B) DHM alleviated TNF‐α‐induced muscle atrophy was dependent on CaMKK, as shown by the immunofluorescence results. Blue represents the nucleus, and red represents atrogin‐1. (C) DHM regulated the protein level of inflammatory response‐induced muscle atrophy‐related genes was dependent on CaMKK, as shown by WB. “p‐” before the gene name means phosphorylated form. (D) DHM regulated the mRNA level of inflammatory response‐induced muscle atrophy‐related genes was dependent on CaMKK, as shown by qPCR. (E) LKB1 was interfered with by small RNA, as shown by qPCR. (F) LKB1 interference did not affect the AMPK expression, as shown by WB. “p‐” before the gene name means phosphorylated form. (F) LKB1 did not affect DHM improvement on TNF‐α‐induced insulin resistance, as shown by the glucose uptake test. (G) LKB1 did not affect DHM improvement on TNF‐α‐induced muscle atrophy, as shown by the immunofluorescence results. Blue represents the nucleus, and red represents atrogin‐1. *N* = 6, ** *p* < 0.01. Bars with different letters indicate they are significantly different (*p *< 0.05)

We blocked the AMPK upstream factors CaMKK and LKB1 to investigate the pathway DHM used to activate AMPK. When STO‐609 blocked CaMKK activity, the ability of DHM to improve TNF‐α‐induced insulin resistance disappeared, as shown by the glucose uptake assay (Figure 7A and Figure S6A). The ability of DHM to prevent TNF‐α‐induced muscle atrophy disappeared after CaMKK was blocked, as shown by the immunofluorescence results (Figure 7B and Figure S6B). When STO‐609 blocked CaMKK activity, the activation effect of DHM on CaMKK, AMPK and mTOR disappeared (Figure 7C and Figure S6C), and the promotion effect of DHM on the expression of IRS and myosin also disappeared (Figure 7C, D and Figure S6C). Blocked CaMKK also caused DHM to lose its inhibitory effect on NF‐κB (Figure 7C, D and Figure S6C); the DHM inhibitory effect on atrogin‐1 expression was also eliminated (Figure 7C, D and Figure S6C). We then interfered with LKB1 expression using siRNA (Figure 7E, F and Figure S6D). LKB1 interference did not affect the activation of AMPK by DHM (Figure 7F and Figure S6D). LKB1 interference did not affect the improvement of DHM on TNF‐α‐induced insulin resistance, as shown by the glucose uptake assay (Figure 7G and Figure S6E). LKB1 interference also did not affect the DHM ability in inhibiting TNF‐α‐induced muscle atrophy, as demonstrated by the immunofluorescence results (Figure 7H and Figure S6F). These results indicated that DHM resisted inflammation‐induced muscle atrophy in C2C12 cells through CaMKK‐AMPK instead of the LKB1‐AMPK pathway.

### 3.8 DHM activated CaMKK by increasing intracellular Ca^2+^ concentration through ryanodine receptor

**FIGURE 8:**
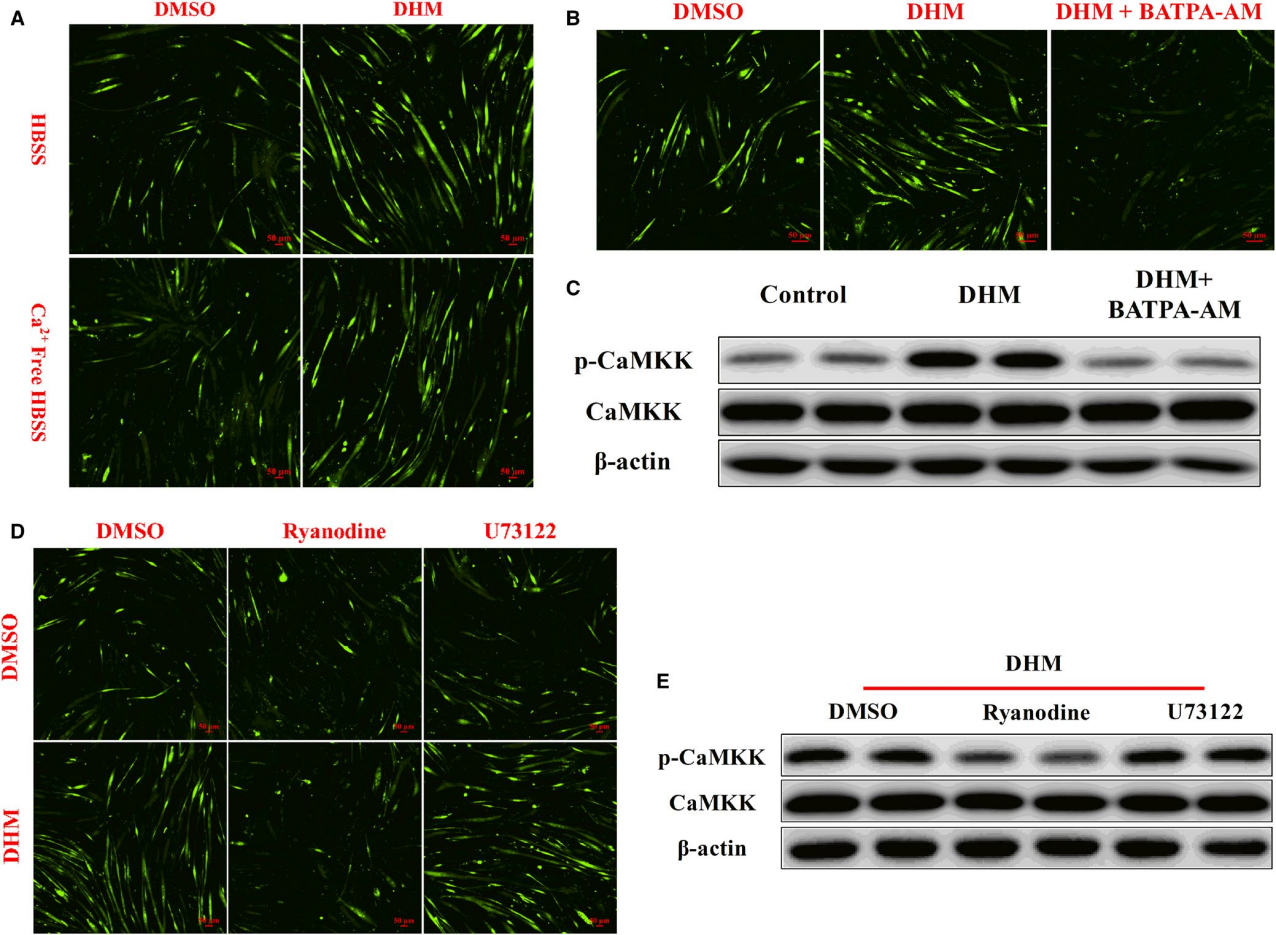
DHM (3 μM) activated CaMKK by increasing intracellular Ca^2+^ concentration through the ryanodine receptor. (A) DHM increased intracellular Ca^2+^ signal, as shown by the Ca^2+^ probe test. (B) After cellular Ca^2+^ was cleared, the activation effect of DHM on intracellular Ca^2+^ was eliminated, as shown by the Ca^2+^ probe test. (C) The effect of DHM on CaMKK activation depended on intracellular Ca^2+^ signal, as shown by WB. (D) DHM increased intracellular Ca^2+^ concentration was dependent on the ryanodine receptor, as shown by the Ca^2+^ probe test. (E) The effect of DHM on CaMKK activation depended on intracellular ryanodine receptor, as shown by WB. *N* = 6

CaMKK was regulated by cellular Ca^2+^, so we investigated whether DHM activated cellular Ca^2+^ signal. Compared with the control group, DHM treatment activated intracellular Ca^2+^ signal, as shown by the Ca^2+^ probe test. When Ca^2+^ was removed from the extracellular medium, DHM still increased intracellular Ca^2+^ level compared with the control group (Figure 8A and Figure S7A). After the cellular Ca^2+^ was cleared by the intracellular Ca^2+^‐chelating agent BATAP‐AM, the activation effect of DHM on intracellular Ca^2+^ was eliminated, indicating that DHM activation on intracellular Ca^2+^ was dependent on intracellular Ca^2+^ storage (Figure 8B and Figure S7B). After cellular Ca^2+^ was cleared, DHM could no longer activate CaMKK, as shown by the WB results (Figure 8C and Figure S7C). Since endoplasmic reticulum was the major organelle for Ca^2+^ release and recovery in myoblasts, we used ryanodine to block the endoplasmic reticulum Ca^2+^ channel ryanodine receptor and U73122 to block the IP3 receptor. Blocking the IP3 receptor pathway did not affect cellular activation of intracellular Ca^2+^ signal induced by DHM (Figure 8D and Figure S7D). After ryanodine blocked the ryanodine receptor, the DHM function on activating intracellular Ca^2+^ signal disappeared (Figure 8D and Figure S7D). After blocking the ryanodine receptor, DHM failed to activate CaMKK, as shown by the WB results (Figure 8E and Figure S7E). These results indicated that DHM activated CaMKK by increasing intracellular Ca^2+^ concentration through the ryanodine receptor.

### 3.9 DHM resisted inflammatory‐induced muscle atrophy through interaction with ryanodine receptor

**FIGURE 9:**
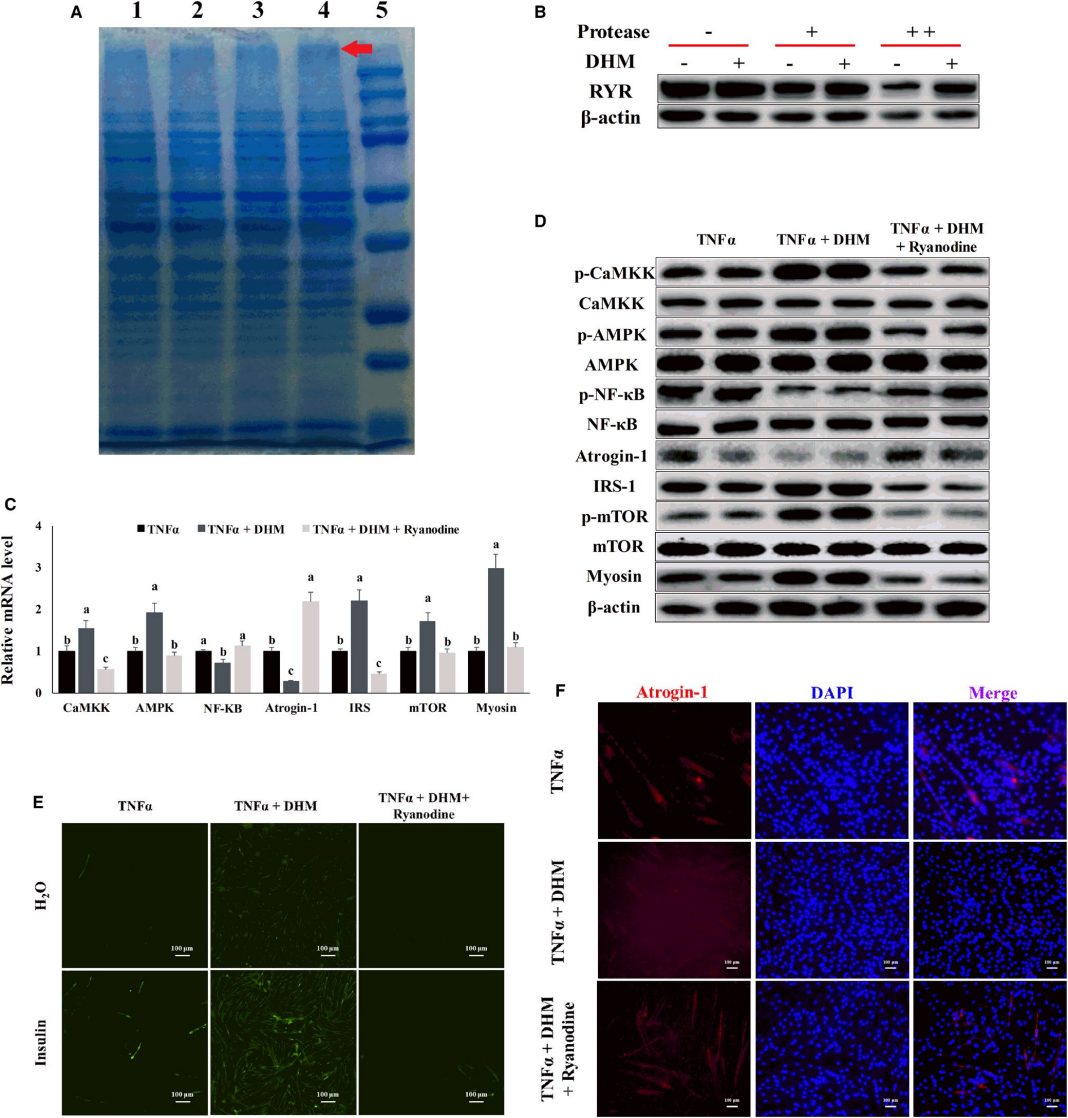
DHM (3 μM) resisted inflammatory‐induced muscle atrophy through interaction with the ryanodine receptor. (A) DHM might interact with the ryanodine receptor, as shown by DARTS, lines 1 to 5 represent H_2_O, DMSO, 10 nM DHM, 1 μM DHM and molecular weight markers, respectively. (B) DHM promoted the stability of ryanodine receptor protein, as shown by WB. (C) The effect of DHM on the mRNA level of inflammatory response‐induced muscle atrophy‐related genes was dependent on the ryanodine receptor, as shown by qPCR. (D) The effect of DHM on the protein level of inflammatory response‐induced muscle atrophy‐related genes was dependent on the ryanodine receptor, as shown by WB. “p‐” before the gene name means phosphorylated form. (E) DHM alleviated TNF‐α‐induced insulin resistance was dependent on the ryanodine receptor, as shown by the glucose uptake test. (F) DHM alleviated TNF‐α‐induced muscle atrophy was dependent on the ryanodine receptor, as shown by the immunofluorescence results. Blue represents the nucleus, and red represents atrogin‐1. *N* = 6. Bars with different letters indicate they are significantly different (*p *< 0.05)

We verified the interaction of DHM and ryanodine receptor by drug affinity responsive target stability (DARTS) assay to investigate the pathway DHM used to activate the ryanodine receptor. From the DARTS results, we found a brighter band at the position of greater than 250 KD SDS‐PAGE in the DHM treatment group compared with the control group, suggesting that DHM might interact with the ryanodine receptor (Figure 9A). At the same time, we also detected the DARTS product through WB. DHM promoted the stability of ryanodine receptor protein compared with the control group, as shown by the WB results, indicating that DHM interacted with ryanodine receptor protein (Figure 9B). When ryanodine blocked the ryanodine receptor, the activation effect of DHM on CaMKK, AMPK and mTOR disappeared (Figure 9C, D and Figure S8A), and the promotion effect of DHM on the expression of IRS and myosin also disappeared (Figure 9C, D and Figure S8A). Blocked ryanodine receptor also caused DHM to lose its inhibitory effect on NF‐κB (Figure 9C, D and Figure S8A); the DHM inhibitory effect on atrogin‐1 expression was also eliminated (Figure 9C, D and Figure S8A). When ryanodine blocked the ryanodine receptor, the ability of DHM to improve TNF‐α‐induced insulin resistance disappeared, as shown by the glucose uptake assay (Figure 9E and Figure S8B). The ability of DHM to prevent TNF‐α‐induced muscle atrophy disappeared after the ryanodine receptor was blocked, as shown by the immunofluorescence results (Figure 9F and Figure S8C). These results indicated that DHM resisted inflammatory‐induced muscle atrophy through interaction with the ryanodine receptor.

## 4 DISCUSSION

Skeletal muscle plays a pivotal role in the maintenance of physical and metabolic health.^21^ Skeletal muscle atrophy, characterized by muscle mass loss and function decline, is due to an increase of muscle protein degradation and reduction of protein synthesis. Muscle mass loss is frequently associated with inflammation, ageing, injury and obesity.^2^ Therefore, therapeutic strategies for skeletal muscle atrophy need to block protein degradation and increase protein synthesis. In this study, we found that DHM activated the Ca^2+^‐CaMKK‐AMPK signal pathway by binding to the ryanodine receptor. Activation of AMPK increased protein synthesis by improving inflammation‐induced skeletal muscle insulin resistance. AMPK also inhibited protein degradation by blocking the inflammation‐induced skeletal muscle inflammatory response. Our study not only clarified the molecular mechanism of DHM resistance to inflammation‐induced skeletal muscle atrophy but also discovered the target of DHM in this process. This study provided a new therapeutic strategy for the obesity‐induced skeletal muscle atrophy.

The increasing incidence of skeletal muscle atrophy is inextricably related to the increase in the global obese population.^22^ A recent report estimated that 2.1 billion people over the world, nearly 30% of the world population, were obese or overweight. During the process of obesity, the proliferation and hypertrophy of adipocytes induced hypoxic stress. Hypoxic stress caused the secretion of inflammatory factors, which induced chronic systemic inflammation.^23^ Our results showed that levels of serum and skeletal muscle inflammatory factors were significantly increased in HFD‐induced obese mice. Inflammatory factors produced by adipose tissue were transported to skeletal muscle and activated NF‐κB signalling. NF‐κB is one of the most important nuclear transcription factors. Under normal circumstances, NF‐κB and IκB form a complex to stay in the cytoplasm. When TNF‐α induced the activation of the IκB kinase (IKK) complex, the IKK triggered the phosphorylation and proteasome degradation of IκB.

Degradation of IκB resulted in the NF‐κB translocation into the nucleus, where NF‐κB induced the expression of skeletal muscle atrophy‐related genes and other inflammatory cytokines^24^, ^25^. Results from us in vivo experiment showed that skeletal muscle inflammatory factor activated the phosphorylation of IκB and NF‐κB and increased the expression of skeletal muscle atrophy‐related genes. Results from us in vitro experiment also showed that TNF‐α induced NF‐κB activation and the expression of muscle atrophy‐associated genes in C2C12 cells. When IRS‐1 serine 307 was phosphorylated by IKK in the NF‐κB signalling pathway and JNK‐1 in the MAPK signalling pathway, PI3K/AKT activation was blocked. Both inflammatory factors and free fatty acids could activate JNK‐1 and NF‐κB directly and then caused insulin resistance.^26^ Results from us in vivo experiments showed that skeletal muscle inflammatory factor activated the JNK‐1 and NF‐κB and induced mice insulin resistance. Results from us in vitro experiments also showed that TNF‐α induced the NF‐κB and JNK‐1 activation and insulin resistance in C2C12 cells.

AMPK is not only a key regulator in cellular energy metabolism but also plays an essential regulatory role in many physiological processes such as inflammation, tumour growth and enhanced insulin sensitivity.^27^ The pharmacological function of DHM to activate AMPK has been elucidated in various physiological processes. DHM (200 mg/kg) protected rats from developing Alzheimer's disease via the up‐regulation of the AMPK/SIRT1 pathway to inhibit inflammatory responses and apoptosis of hippocampal cells and ameliorate cognitive function.^28^ DHM improved skeletal muscle insulin sensitivity partially through inducing autophagy by activating the AMPK‐PGC‐1α‐Sirt3 signalling pathway.^29^ We demonstrated that DHM activated AMPK in vivo and in vitro, and we also showed that the function of DHM in blocking muscle atrophy depended on its activation effect on AMPK in vitro.

As the key regulator of cellular energy metabolism, AMPK inhibited anabolism to and promoted catabolism.^30^ In this study, we found that DHM alleviated the levels of glucose and free fatty acids in serum caused by HFD. We also found that DHM increased oxygen consumption and decreased respiratory exchange ratio without affecting exercise frequency. The consumption of oxygen reflected animal metabolic strength. The respiratory exchange ratio is 1 when the carbohydrates are the only substrate, while the respiratory exchange ratio is about 0.7 when fatty acids are the only substrate.^31^ Our results indicated that DHM not only increased the level of basal metabolism in mice but also increased the proportion of fatty acids in the energy substrate.

AMPK not only regulated energy metabolism but also blocked the inflammatory response by inhibiting the NF‐κB signalling pathway. After AMPK activation, SIRT1 was activated by the lowered NAD^+^ level. P65, the subunit of NF‐κB, was deacetylated by SIRT1; then, the transcriptional activity of NF‐κB was also inhibited.^32^ Inflammatory cytokines and fatty acids activated NF‐κB signalling through binding to the cell membrane Toll‐like receptor 4. Activation of AMPK by agonist blocked IKK and NF‐κB phosphorylation directly.^33^ In this study, we found that DHM blocked NF‐κB phosphorylation and prevented muscle atrophy and insulin resistance through AMPK in vivo and in vitro.

To our knowledge, most DHM‐related research reported that DHM functions depend on its activation of AMPK,^34^ but AMPK is not the direct target of DHM in cells, and the target of DHM in cells is still unclear. Two predominant upstream kinases are known to activate AMPK: LKB1 and CaMKK.^35^ Zyflamend, a blend of herbal extracts, effectively inhibited castrate‐resistant prostate tumour growth through activating the LKB1‐AMPK signal pathway.^36^ Eugenol effectively ameliorated hyperglycaemia by inhibiting hepatic gluconeogenesis via modulating the CaMKK‐AMPK‐CREB signalling pathway.^37^ To investigate the pathway DHM used to activate AMPK, we blocked the AMPK upstream factors CaMKK and LKB1. Results from us in vitro experiment showed that DHM resisted inflammation‐induced muscle atrophy through CaMKK‐AMPK instead of the LKB1‐AMPK pathway.

As far as we know, there is no published report on how DHM activates CaMKK. Brosimone I, an isoprenoid‐substituted flavonoid from Artocarpus heterophyllus, induced endoplasmic reticulum stress leading to cytosolic Ca^2+^ increase. Brosimone I induced cell cycle G1 phase arrest and apoptosis via the activation of the Ca^2+^‐CaMKK‐AMPK signalling pathway.^38^ Zearalenone increased cellular Ca^2+^ release by inducing endoplasmic reticulum stress. Zearalenone induced autophagy by activating the Ca^2+^‐CaMKK‐AMPK signalling pathway.^39^ These reports indicated that plant extracts could activate the CaMKK‐AMPK signalling pathway through Ca^2+^. In this study, we found that the DHM activated CaMKK‐AMPK signalling pathway is dependent on Ca^2+^.

Dandelion root extract (10–400 µg/ml) dose dependently increased intracellular Ca^2+^ level in the presence of external Ca^2+^. The dandelion root extract‐induced Ca^2+^ increase was significantly reduced in the absence of extracellular Ca^2+^.^40^ However, in our study, DHM activated intracellular Ca^2+^ signals regardless of the presence or absence of extracellular Ca^2+^. The endoplasmic reticulum is the main storage organelle for calcium ion in skeletal muscle. The ryanodine receptor and IP3 receptor are the main channels for calcium release from the endoplasmic reticulum and play a central role in excitation‐contraction coupling in skeletal muscle.^41^ In this study, we discovered that blocking the ryanodine receptor, the DHM’s ability to activate Ca^2+^ and CaMKK disappeared, but blocking the IP3 receptor had no effect on preventing the activation of Ca^2+^‐CaMKK induced by DHM.

The identification of target protein for small molecules is critical for chemical metabolomics and drug discovery.^42^ DARTS is a straightforward and quick approach to identify potential target protein of small molecules. The mechanism of DARTS is that the interaction of target protein and small molecules resists proteolysis. The most significant advantage of this method is it allows the use of the small native molecule without any immobilization or modification, such as the incorporation of biotin, radioisotope or photo‐affinity labels.^20^, ^43^ In this study, we used DARTS to identify potential binding targets of DHM and used DARTS‐Western blotting to test and validate the potential DHM target. Our results showed that DHM interacted with ryanodine receptor protein to activate the Ca^2+^‐CaMKK‐AMPK signal pathway.

**FIGURE 10:**
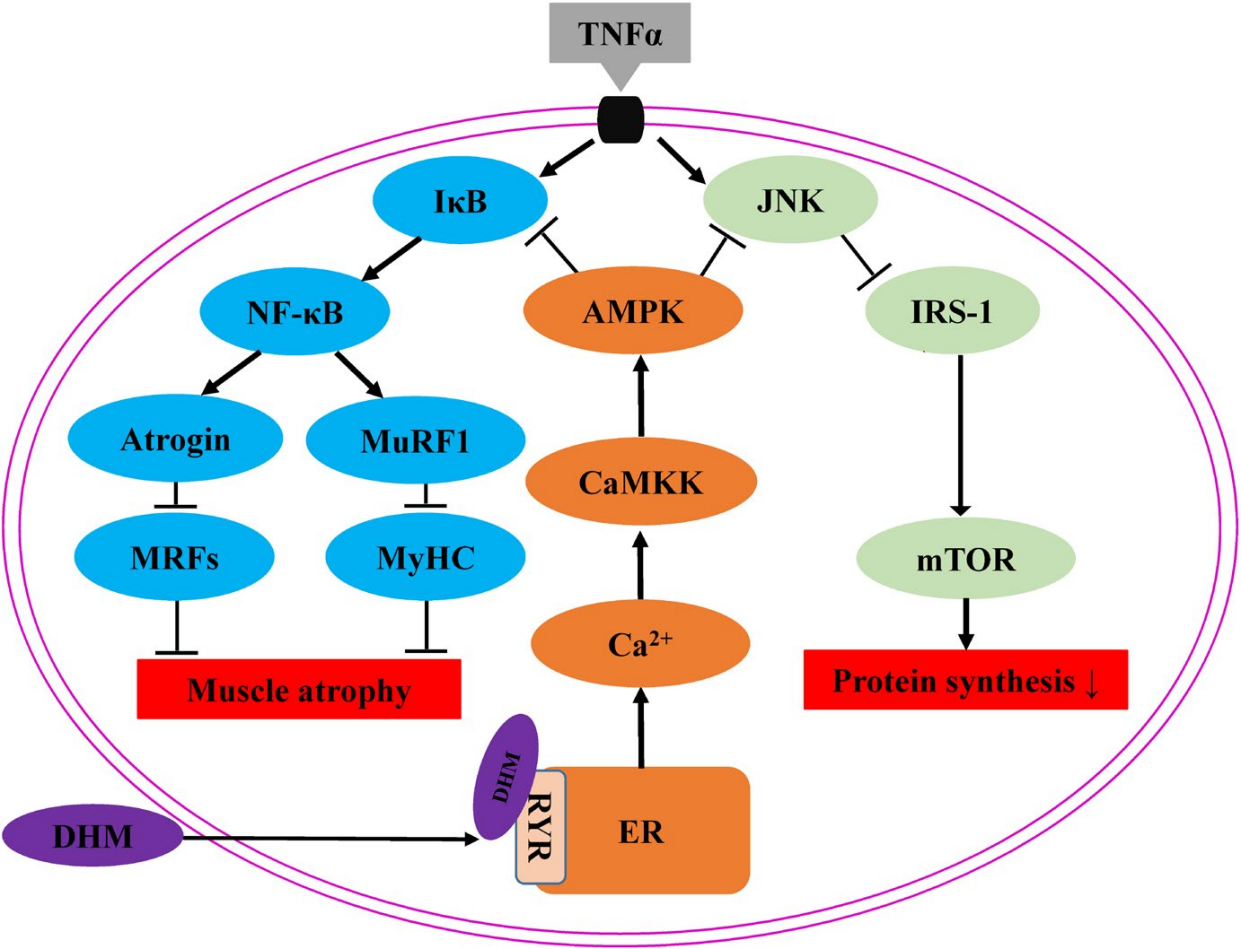
Summary model of dihydromyricetin resists inflammation‐induced muscle atrophy via the ryanodine receptor‐CaMKK‐AMPK signal pathway

In conclusion, our results not only show that DHM resisted inflammation‐induced muscle atrophy but also demonstrate that the DHM activated the Ca^2+^‐CaMKK‐AMPK signal pathway through interacting with its target protein ryanodine receptor (Figure 10). Our results provided experimental data for the development of DHM as a functional food and new therapeutic strategies for treating or preventing skeletal muscle atrophy.

## CONFLICT OF INTEREST

The authors declare that there are no known conflicts of interest associated with this publication and there has been no significant financial support for this work that could have influenced its outcome.

## AUTHOR CONTRIBUTIONS

**Lianjie Hou:** Conceptualization (equal); Writing‐review & editing (equal). **Fangyi Jiang:** Investigation (equal); Resources (equal). **Bo Huang:** Software (equal); Visualization (equal). **Weijie Zheng:** Investigation (equal); Methodology (equal). **Yufei Jiang:** Investigation (equal); Methodology (equal). **Gengyuan Cai:** Resources (equal). **Dewu Liu:** Funding acquisition (equal); Resources (equal). **Chingyuan Hu:** Conceptualization (equal); Writing‐review & editing (equal). **Chong Wang:** Conceptualization (equal); Funding acquisition (equal); Investigation (equal); Writing‐review & editing (equal).

## CONSENT FOR PUBLICATION

All authors have read and approved for publication.

## Supporting information

Fig S1‐S8

## Data Availability

We confirm that the data supporting the findings of this study are available within the article and its supplementary materials.
