# Supplementary material for: Dihydromyricetin resists inflammation‐induced muscle atrophy via ryanodine receptor‐CaMKK‐AMPK signal pathway
Source: J Cell Mol Med. 2021 Oct 22;25(21):9953–71. doi: 10.1111/jcmm.16810 (PMC8572760; doi:10.1111/jcmm.16810)
Supplement: Supplementary file 1 — Fig S1‐S8 [file JCMM-25-9953-s001.docx]

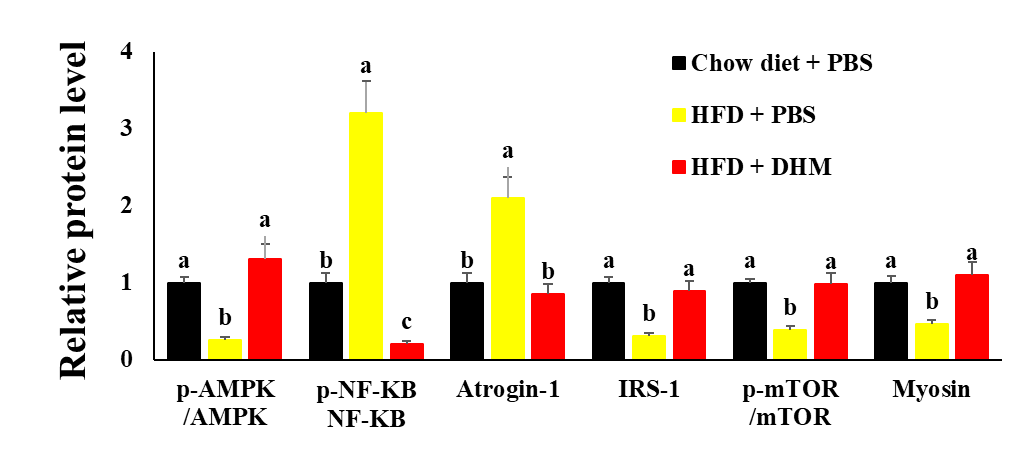


Figure S1. Grayscale scanning statistics of inflammatory induced skeletal muscle atrophy-related proteins in Figure 2I. N = 6. Bars with different letters indicate they are significantly different (P<0.05).


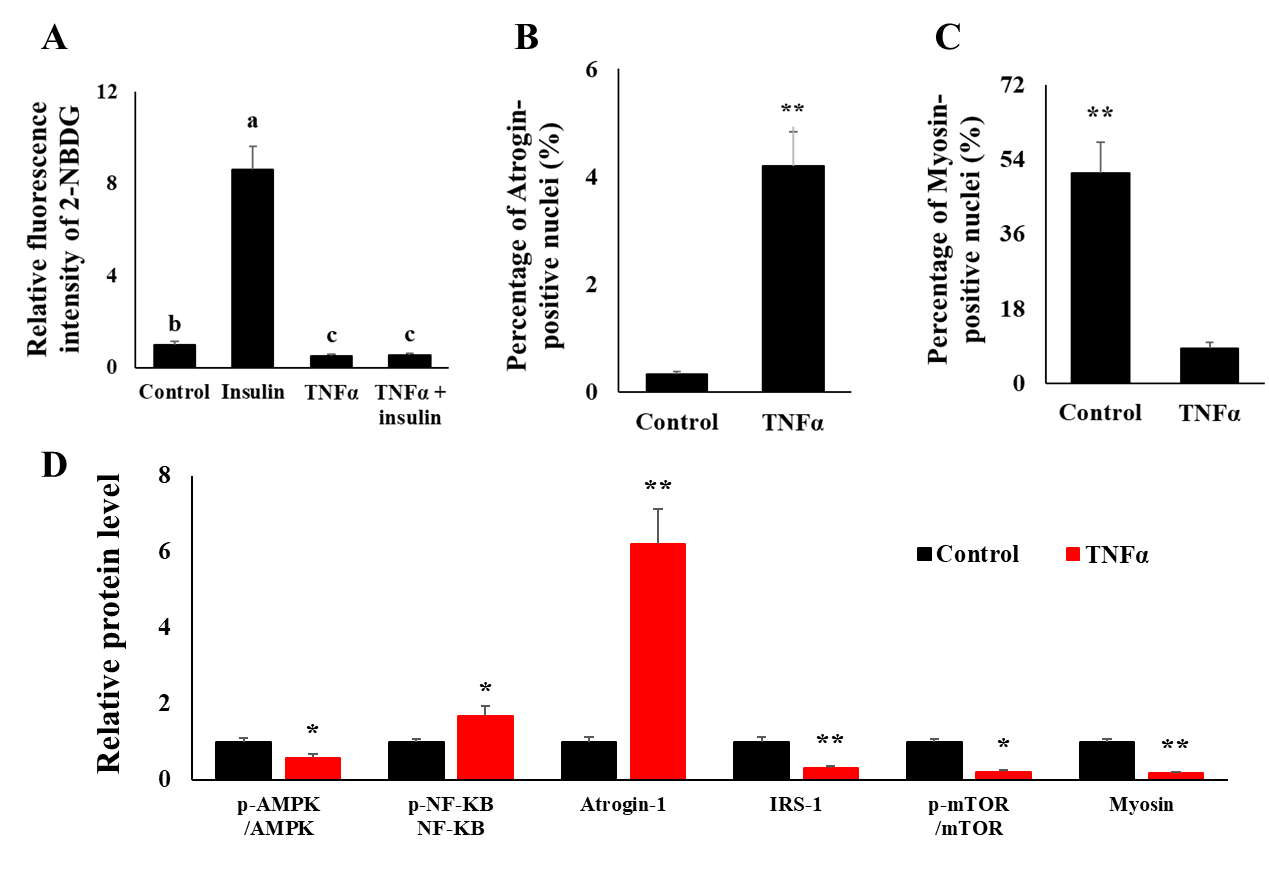


Figure S2. Statistical results related to Figure 3. A. Statistical results related to the relative fluorescence intensity of 2-NBDG in Figure 3B. B. Statistical results of Atrogin-1 immunofluorescence results in Figure 3C. C. Statistical results of Myosin immunofluorescence results in Figure 3D. D. Grayscale scanning statistics of inflammatory induced skeletal muscle atrophy-related proteins in Figure 3E. N = 6, * *p* < 0.05, ** *p* < 0.01. Bars with different letters indicate they are significantly different (*P*<0.05).


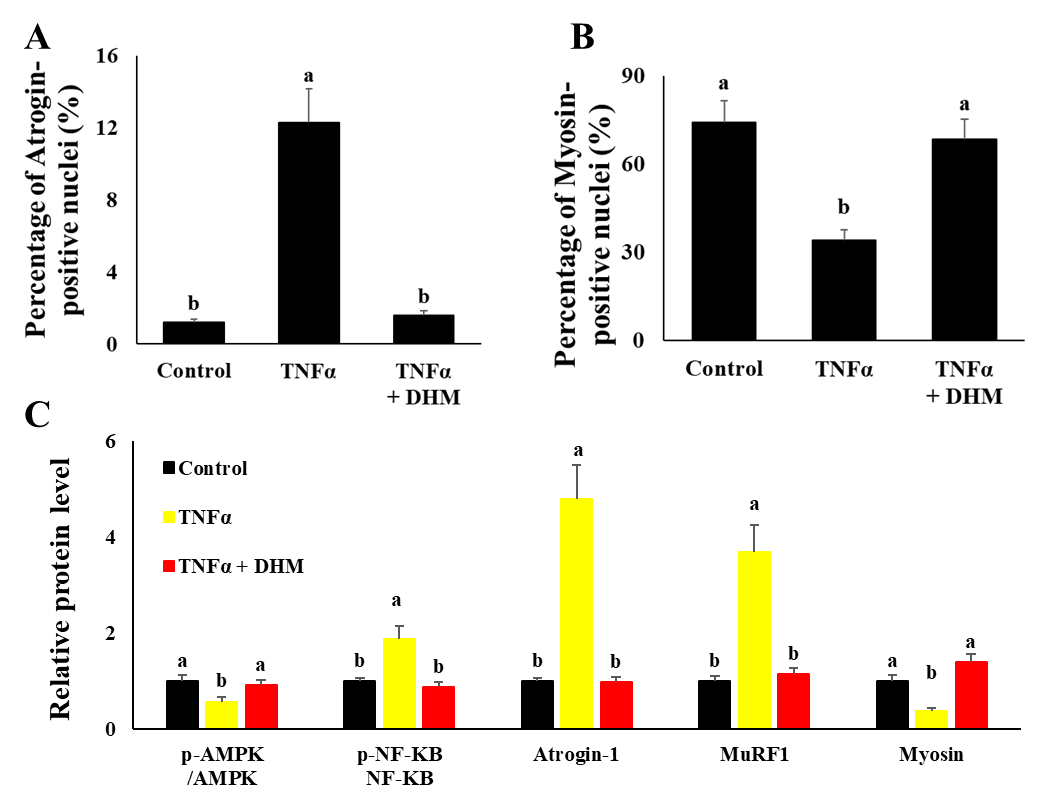


Figure S3. Statistical results related to Figure 4. A. Statistical results of Atrogin-1 immunofluorescence results in Figure 4B. B. Statistical results of Myosin immunofluorescence results in Figure 4C. C. Grayscale scanning statistics of inflammatory induced skeletal muscle atrophy-related proteins in Figure 4E. N = 6. Bars with different letters indicate they are significantly different (*P*<0.05).


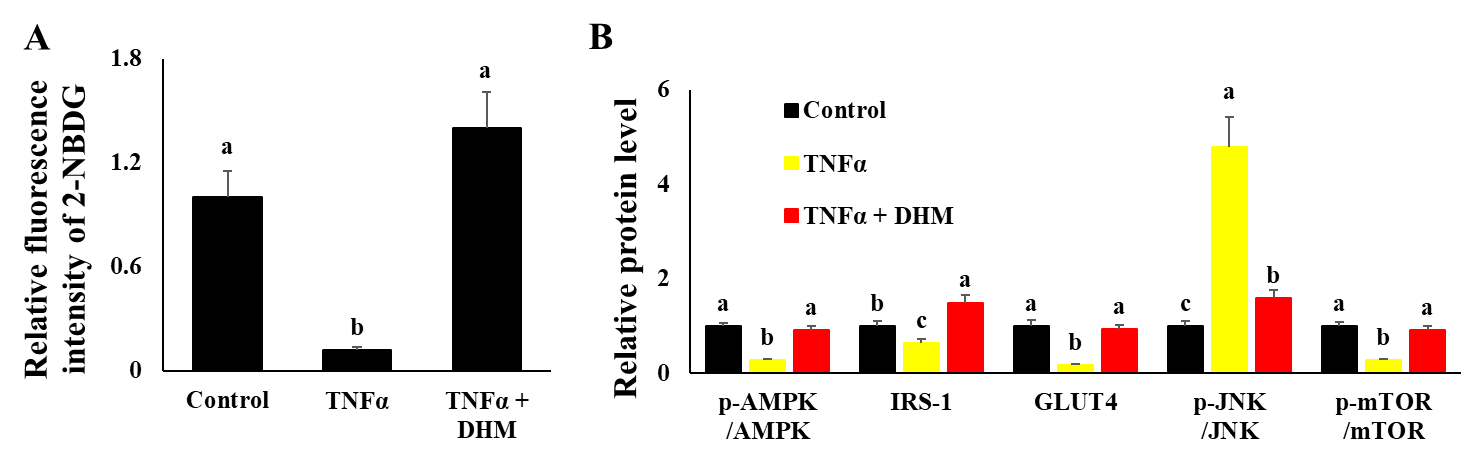


Figure S4. Statistical results related to Figure 5. A. Statistical results related to the relative fluorescence intensity of 2-NBDG in Figure 5A. B. Grayscale scanning statistics of insulin resistance and protein synthesis-related proteins in Figure 5C. N = 6. Bars with different letters indicate they are significantly different (*P*<0.05).


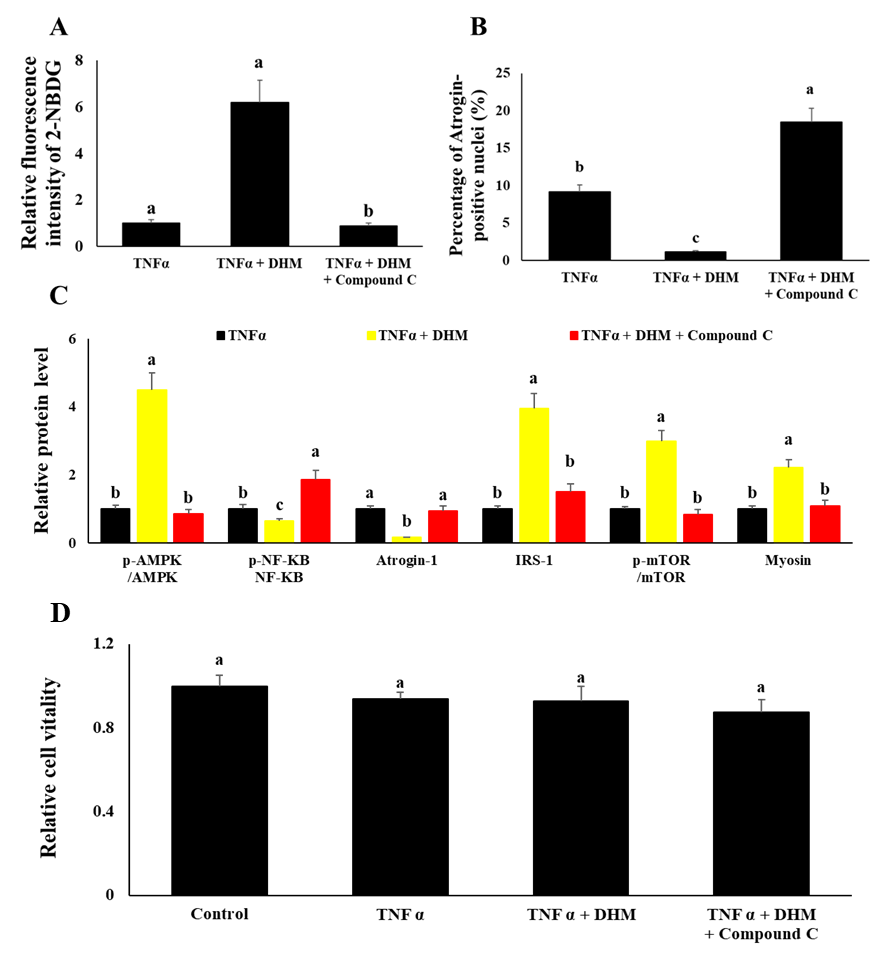


Figure S5. Statistical results related to Figure 6. A. Statistical results related to the relative fluorescence intensity of 2-NBDG in Figure 6A. B. Statistical results of Atrogin-1 immunofluorescence results in Figure 6B. C. Grayscale scanning statistics of inflammatory induced skeletal muscle atrophy-related proteins in Figure 6C. D. The treatments on C2C12 cell vitality was determined by MTT. N = 6. Bars with different letters indicate they are significantly different (*P*<0.05).


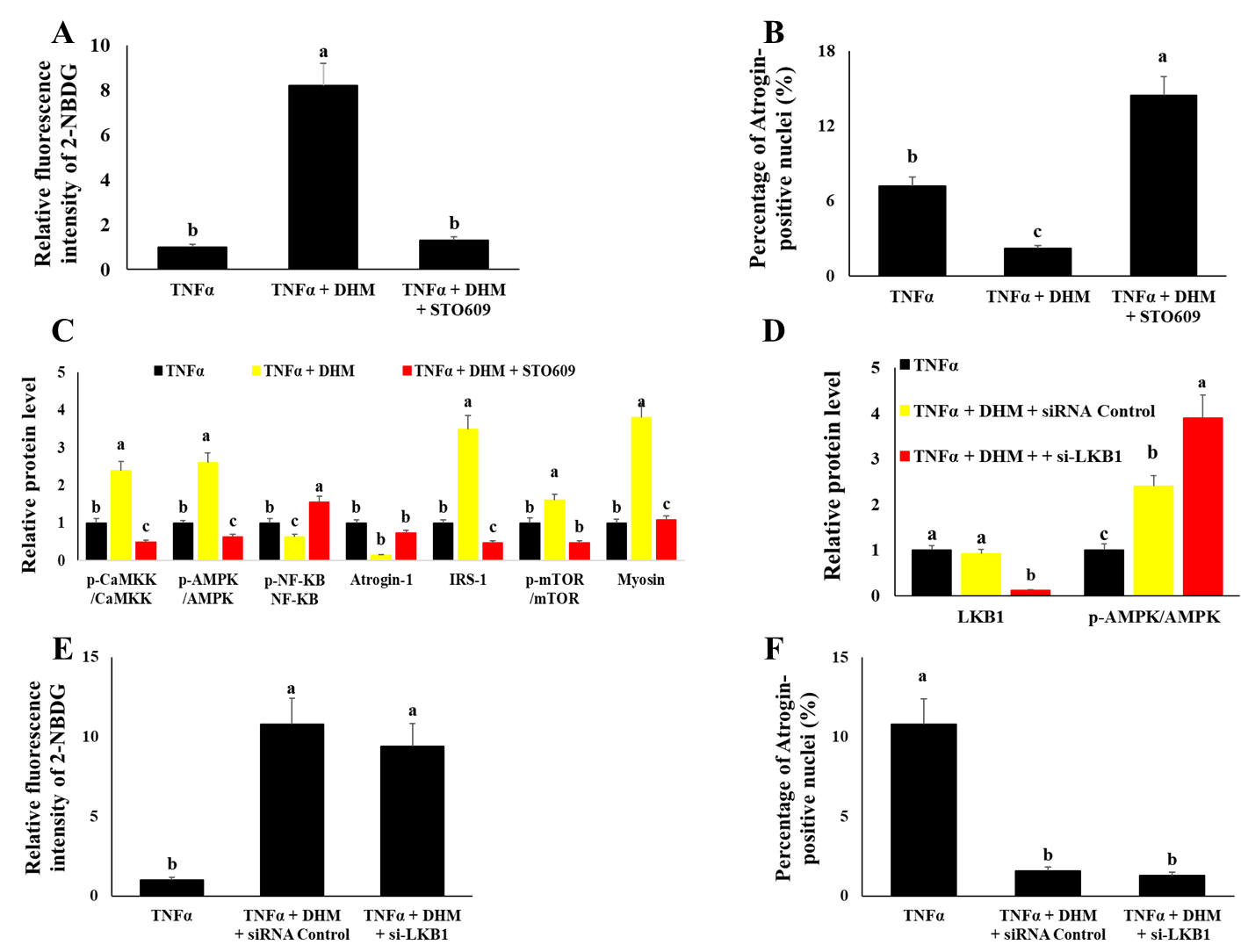


Figure S6. Statistical results related to Figure 7. A. Statistical results related to the relative fluorescence intensity of 2-NBDG in Figure 7A. B. Statistical results of Atrogin-1 immunofluorescence results in Figure 7B. C. Grayscale scanning statistics of inflammatory induced skeletal muscle atrophy-related proteins in Figure 7C. D. Grayscale scanning statistics of LKB1 and p-AMPK proteins in Figure 7F. E. Statistical results related to the relative fluorescence intensity of 2-NBDG in Figure 7G. F. Statistical results of Atrogin-1 immunofluorescence results in Figure 7H. N = 6. Bars with different letters indicate they are significantly different (*P*<0.05).


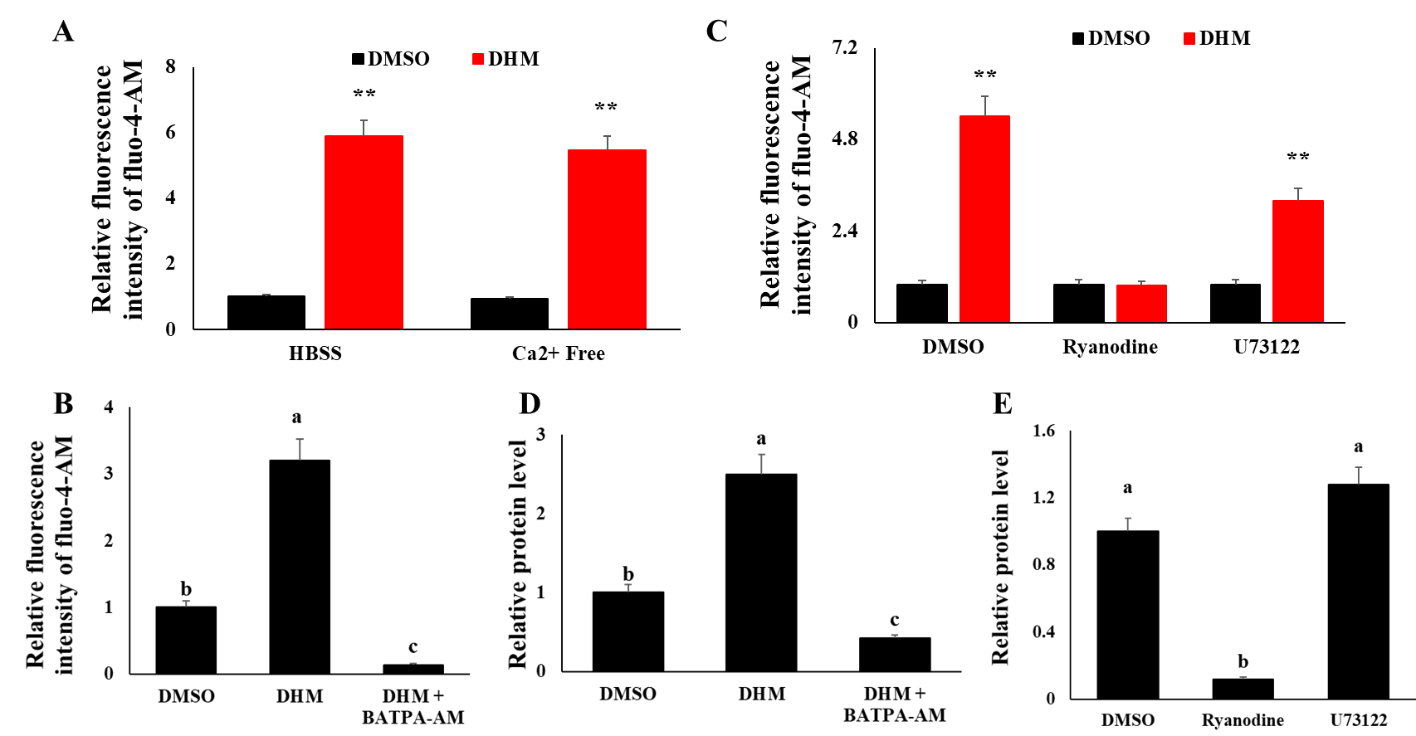


Figure S7. Statistical results related to Figure 8. A. Statistical results related to the relative fluorescence intensity of Fluo-4-AM in Figure 8A. B. Statistical results related to the relative fluorescence intensity of Fluo-4-AM in Figure 8B. C. Statistical results related to the relative fluorescence intensity of Fluo-4-AM in Figure 8C. D. Grayscale scanning statistics of CaMKK proteins in Figure 8D. E. Grayscale scanning statistics of CaMKK proteins in Figure 8E. N = 6. Bars with different letters indicate they are significantly different (*P*<0.05).


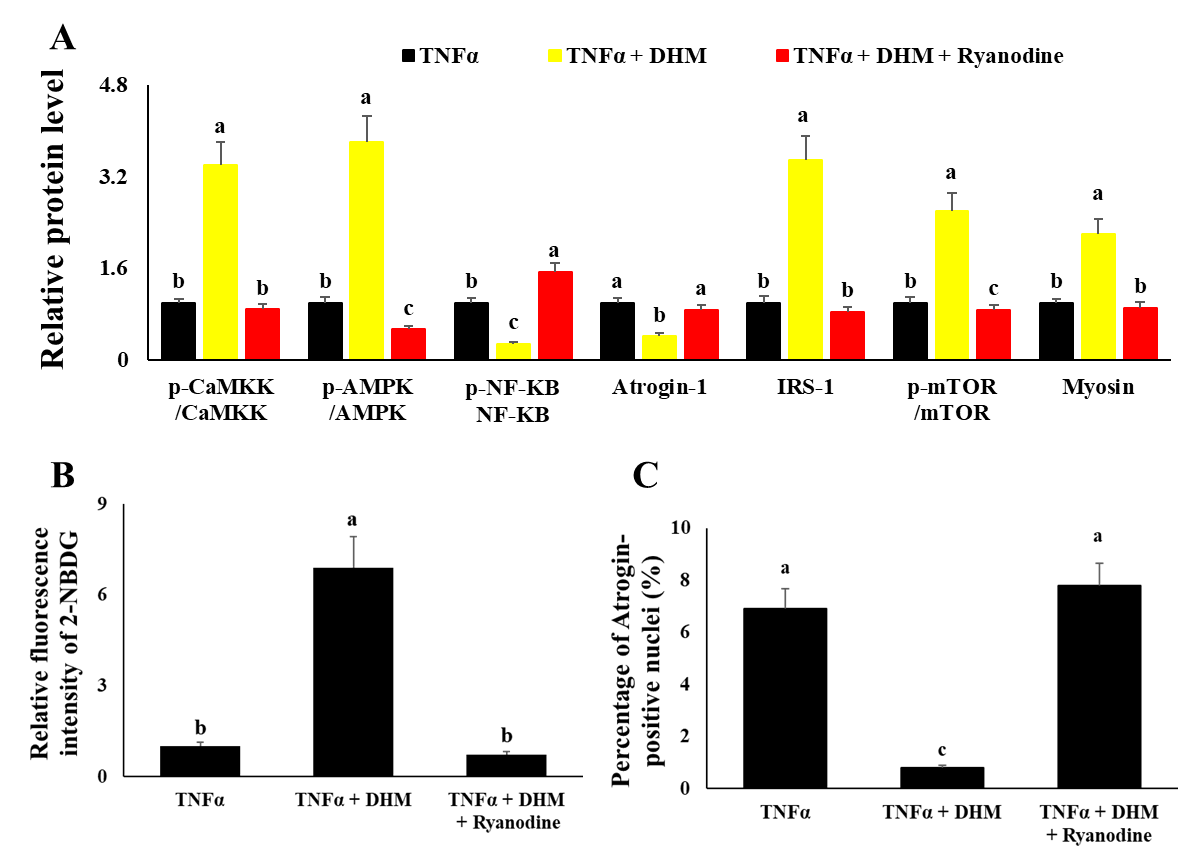


Figure S8. Statistical results related to Figure 9. A. Grayscale scanning statistics of inflammatory induced skeletal muscle atrophy-related proteins in Figure 9D. B. Statistical results related to the relative fluorescence intensity of 2-NBDG in Figure 9E. C. Statistical results of Atrogin-1 immunofluorescence results in Figure 9F. N = 6. Bars with different letters indicate they are significantly different (*P*<0.05).
